# From an antiferromagnetic insulator to a strongly correlated metal in square-lattice MCl_2_(pyrazine)_2_ coordination solids

**DOI:** 10.1038/s41467-022-33342-5

**Published:** 2022-09-30

**Authors:** Panagiota Perlepe, Itziar Oyarzabal, Laura Voigt, Mariusz Kubus, Daniel N. Woodruff, Sebastian E. Reyes-Lillo, Michael L. Aubrey, Philippe Négrier, Mathieu Rouzières, Fabrice Wilhelm, Andrei Rogalev, Jeffrey B. Neaton, Jeffrey R. Long, Corine Mathonière, Baptiste Vignolle, Kasper S. Pedersen, Rodolphe Clérac

**Affiliations:** 1 Univ. Bordeaux, CNRS, Centre de Recherche Paul Pascal, CRPP, UMR 5031, 33600 Pessac, France; 2 Univ. Bordeaux, CNRS, Bordeaux INP, ICMCB, UMR 5026, 33600 Pessac, France; 3 Chemistry Faculty, University of the Basque Country, UPV/EHU, 20018 Donostia-San Sebastián, Spain; 4 BCMaterials, Basque Center for Materials, Applications and Nanostructures, UPV/EHU Science Park, 48940 Leioa, Spain; 5 IKERBASQUE, Basque Foundation for Science, 48009 Bilbao, Spain; 6 Department of Chemistry, Technical University of Denmark, 2800 Kgs Lyngby, Denmark; 7 Department of Chemistry, The University of Oxford, Oxford, OX1 3QR UK; 8 Departamento de Ciencias Físicas, Universidad Andres Bello, 837-0136 Santiago, Chile; 9 Department of Chemistry, University of California Berkeley, Berkeley, CA 94720 USA; 10 Univ. Bordeaux, CNRS, Laboratoire Ondes et Matière d’Aquitaine, UMR 5798, 33400 Talence, France; 11 ESRF—The European Synchrotron, 38043 Grenoble, France; 12 Molecular Foundry, Lawrence Berkeley National Laboratory, Berkeley, Berkeley, CA 94720 USA; 13 Department of Physics, The University of California, Berkeley, Berkeley, CA 94720 USA; 14 Kavli Energy Nanosciences Institute at Berkeley, Berkeley, CA 94720 USA; 15 Department of Chemical and Biomolecular Engineering, University of California Berkeley, Berkeley, CA 94720 USA; 16 Materials Sciences Division, Lawrence Berkeley National Laboratory, Berkeley, CA 94720 USA

**Keywords:** Magnetic materials, Coordination chemistry, Inorganic chemistry, Metal-organic frameworks, Magnetic properties and materials

## Abstract

Electronic synergy between metal ions and organic linkers is a key to engineering molecule-based materials with a high electrical conductivity and, ultimately, metallicity. To enhance conductivity in metal-organic solids, chemists aim to bring the electrochemical potentials of the constituent metal ions and bridging organic ligands closer in a quest to obtain metal-*d* and ligand-*π* admixed frontier bands. Herein, we demonstrate the critical role of the metal ion in tuning the electronic ground state of such materials. While VCl_2_(pyrazine)_2_ is an electrical insulator, TiCl_2_(pyrazine)_2_ displays the highest room-temperature electronic conductivity (5.3 S cm^–1^) for any metal-organic solid involving octahedrally coordinated metal ions. Notably, TiCl_2_(pyrazine)_2_ exhibits Pauli paramagnetism consistent with the specific heat, supporting the existence of a Fermi liquid state (i.e., a correlated metal). This result widens perspectives for designing molecule-based systems with strong metal-ligand covalency and electronic correlations.

## Abstract (web summary)

Achieving high conductivity in metal-organic solids can be challenging, due to the difficulty of obtaining a good overlap between the *d*-orbitals of the metal and the *π*-orbitals of the organic molecule. Here, the authors present two coordination solids, VCl_2_(pyrazine)_2_ and TiCl_2_(pyrazine)_2_, with remarkably different electrical conductivity. While the former is an insulator, the latter displays the highest conductivity of any octahedrally coordinated metal ions based metal-organic solid.

## Introduction

The realization of strong conjugation between metal-centered *d*-orbitals and the *π*-orbitals of bridging ligands qualifies metal-organic frameworks (MOFs) and, more generally, coordination solids for diverse applications in spintronics^1–3^, magnetoelectrics^4,5^, or as electrocatalysts^6,7^, sensors^8^, field-effect transistors^9^, and supercapacitors^10^. One of the main factors that currently limits the further development of such applications is the electrically insulating nature of almost all coordination solids^11^. Encouraged by the incomparable possibilities available to modern molecular and coordination chemistries for tuning the physical properties of molecule-based materials, scientists are actively working on lifting this limitation^12^. While organic metals were discovered already in 1973^13^, there are only very few coordination solids displaying clear metallic conductivity. Among them, layered materials, pillared on transition metal ions with square-planar or distorted tetrahedral coordination geometries, have been reported to exhibit metallic properties as well as superconductivity^14–17^. For example, Dincă et al. reported metallicity in the M_3_(hexaiminobenzene)_2_ (M = Ni, Cu), as suggested from ultraviolet photoelectron spectroscopy and band structure calculations^18^. Later, Marinescu and coworkers demonstrated that air-exposure of an iron 2,3,6,7,10,11-triphenylenehexathiolate MOF led to a material featuring a semiconducting-to-metallic phase transition around room temperature^19^. The presence of a metallic state is commonly evidenced experimentally by an increase of the electronic conductivity upon lowering the temperature, as reported for a single crystal of Cu^I^(2,5-DM-DCNQI)_2_ (2,5-DM-DCNQI = 2,5-dimethyl-*N,N’*-dicyanoquinonediimine)^16^. Unfortunately, this straightforward fingerprint of a metallic state is not easy to obtain when the resistivity measurements are performed on pressed polycrystalline or powdered samples for which grain contacts often dominate the charge transport properties^20^. Therefore, metallic states in coordination solids may indeed be more widespread than experimentally demonstrated.

While coordination solids are commonly built with octahedrally coordinated metal ions, their electronic conductivity^21–26^ is found to be lower than for systems based upon square-planar metal-ion nodes, which puts severe limitations on the materials that could be exploited. A significant orbital overlap between the metal-ion centered *d* orbitals and the bridging ligand *π* system is key for engendering high conductivity and critical for designing a metallic state. However, this picture is not theoretically constrained to square-planar coordinated metal ions, but solely relies on orbital symmetries and relative energies^21–26^. We recently reported the coordination solid **CrCl**_**2**_**(pyz)**_**2**_ (pyz = pyrazine) to behave as a semiconductor that could be converted into an insulator upon post-synthetic modification^27,28^. This material, synthesized from Cr^II^ and pyrazine, features octahedrally coordinated Cr centers and exhibits a sizable room-temperature conductivity of 0.032 S cm^–1^. The electrochemical Cr^III^/Cr^II^ reduction potential allows an electron transfer to a single pyrazine ligand during the synthesis leading to Cr^III^ and half-reduced pyrazines (pyz_2_)^–^ in the final material. This partially reduced ligand scaffold, in conjunction with close spatial proximity of spin-carriers, proffers high electronic conductivity by a charge hopping mechanism. Naturally, the conductivity is expected to be strongly dependent on the energy of the *d* orbitals that are involved in the conduction bands at the Fermi level. To this end, the substitution of chromium by other neighboring transition elements (M) provides a route to chemically engineer the electronic conductivity in MCl_2_(pyz)_2_ analogs.

In this work, we demonstrate that the novel **VCl**_**2**_**(pyz)**_**2**_ and **TiCl**_**2**_**(pyz)**_**2**_ coordination solids display contrasting electronic properties. While **VCl**_**2**_**(pyz)**_**2**_ features V^II^ metal centers and a neutral pyrazine scaffold, crystallographic analysis, X-ray absorption spectroscopy, and density functional theory (DFT) studies reveal the presence of Ti^III^ in **TiCl**_**2**_**(pyz)**_**2**_ in conjunction with a one-electron reduced ligand scaffold akin to that of the analoguous **CrCl**_**2**_**(pyz)**_**2**_ material^27^. However, the magnetic properties and electronic conductivity of **VCl**_**2**_**(pyz)**_**2**_ and **TiCl**_**2**_**(pyz)**_**2**_ are strikingly different to those of **CrCl**_**2**_**(pyz)**_**2**_. While **VCl**_**2**_**(pyz)**_**2**_ is a simple antiferromagnetic insulator, both temperature-independent (Pauli) paramagnetism and a residual electronic specific heat term are observed in **TiCl**_**2**_**(pyz)**_**2**_, revealing an unprecedented Fermi liquid metallic state in a coordination solid.

## Results and discussion

### Synthesis, structure, and spectroscopic characterization

**Fig. 1:**
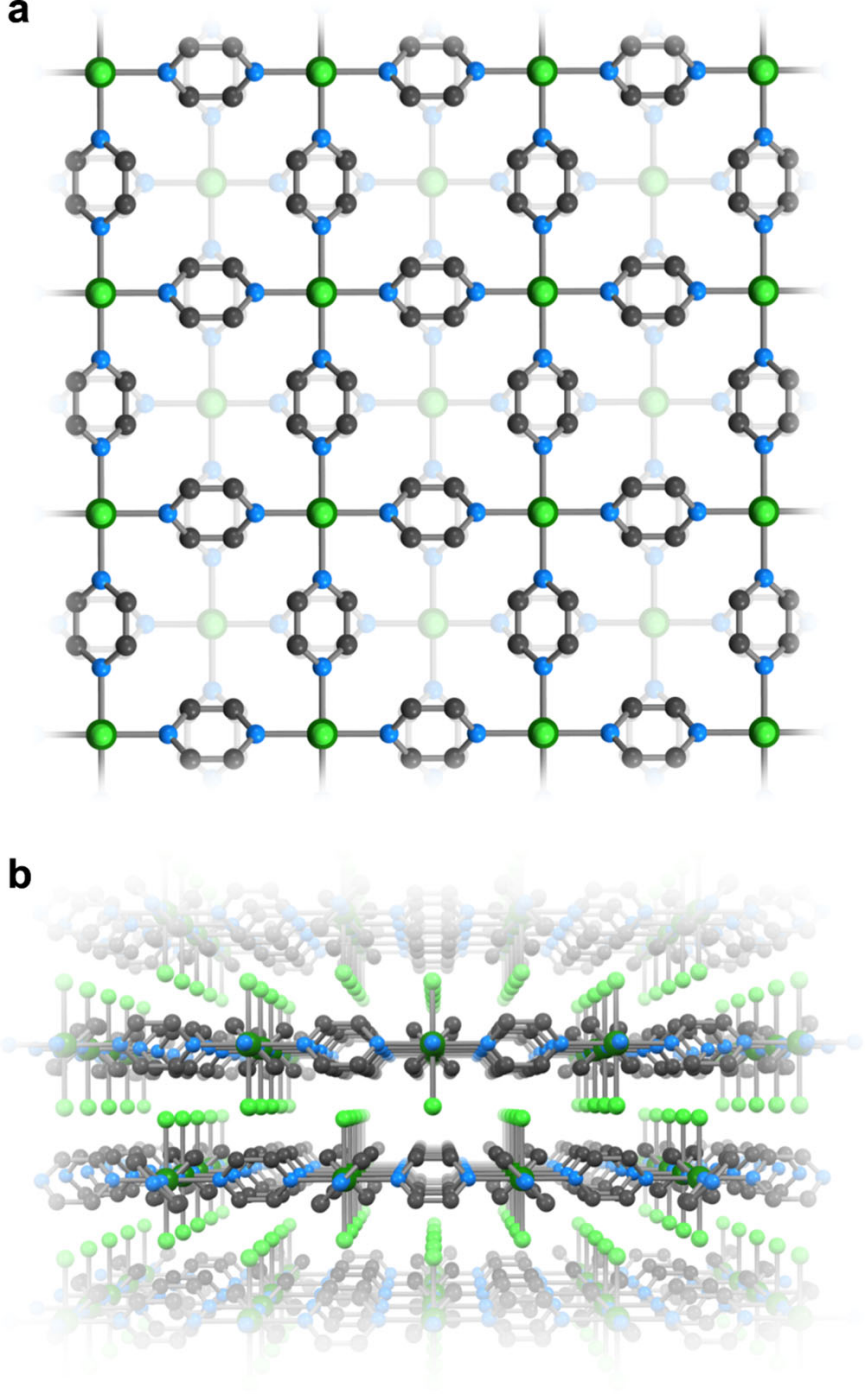
X-ray crystal structure. Structure of **VCl**_**2**_**(pyz)**_**2**_ as determined from single-crystal X-ray diffraction (*T* = 120(1) K) shown perpendicular (**a**) and parallel (**b**) to the 2D layers. Only one orientation of the positionally disordered pyz ligands is shown. Color code: V, dark green; Cl, green; N, blue; C, gray.

The reaction of TiCl_2_ and VCl_2_ with a large excess of pyrazine at 200 °C produces deep black **TiCl**_**2**_**(pyz)**_**2**_ and dark purple **VCl**_**2**_**(pyz)**_**2**_, respectively, as microcrystalline powders (see Methods). Scanning electron microscopy (Supplementary Figs. 1, 2) and Williamson-Hall analysis of the X-ray powder diffractograms (Supplementary Fig. 3) both suggest a higher degree of crystallinity of **VCl**_**2**_**(pyz)**_**2**_ over **TiCl**_**2**_**(pyz)**_**2**_, with micrometer and sub-micrometer size crystallites, respectively. Synchrotron powder X-ray diffraction data at room temperature suggest that **TiCl**_**2**_**(pyz)**_**2**_ and **VCl**_**2**_**(pyz)**_**2**_ are isostructural, and structurally similar to the Cr analog (Supplementary Figs. 3, 4 and Supplementary Table 1). In contrast to **CrCl**_**2**_**(pyz)**_**2**_ (space group *Immm*), **TiCl**_**2**_**(pyz)**_**2**_ and **VCl**_**2**_**(pyz)**_2_ are isomorphous to the previously reported CoCl_2_(pyz)_2_ and NiCl_2_(pyz)_2_ compounds, crystallizing in the tetragonal *I*4/*mmm* space group^29,30^. Their structures consist of a perfectly square lattice of pyrazine-bridged metal centers with two *trans*-coordinated and non-bridging chloride ligands oriented along the crystallographic *c* direction. Single-crystals were obtained only for **VCl**_**2**_**(pyz)**_**2**_ by optimizing the thermal process during the synthesis (see Methods). X-ray crystallographic analysis on a single crystal of **VCl**_**2**_**(pyz)**_**2**_ at 120 K further corroborates the above findings on microcrystalline powders (Fig. 1, Supplementary Fig. 5, Supplementary Table 2). Even at 120 K and due to the symmetry of the *I*4/*mmm* space group, a disorder of the pyrazine ligands over two positions is present. The room-temperature Ti–N and Ti–Cl bond lengths in **TiCl**_**2**_**(pyz)**_**2**_ amount to 2.14 and 2.37 Å, respectively, which are similar to the V–N and V–Cl bond lengths in **VCl**_**2**_**(pyz)**_**2**_ of 2.15 and 2.42 Å (2.14 and 2.42 Å at 120 K), respectively. The molecular compound *trans*-[V^II^Cl_2_(py)_4_] (py = pyridine) closely mimics the local coordination geometry of **VCl**_**2**_**(pyz)**_**2**_ with V–N and V–Cl bond lengths of 2.18 and 2.46 Å, respectively (at 120 K, Supplementary Table 3, Supplementary Fig. 6), suggesting the presence of V^II^ metal ions^31^. On the other hand, the conclusion is not straightforward when comparing the *trans*-[Ti^II^Cl_2_(py)_4_]^32^ complex with 2.17 Å Ti–N and 2.44 Å Ti–Cl bond lengths (at 153 K) with the slightly shorter bonds observed in **TiCl**_**2**_**(pyz)**_**2**_.

**Fig. 2:**
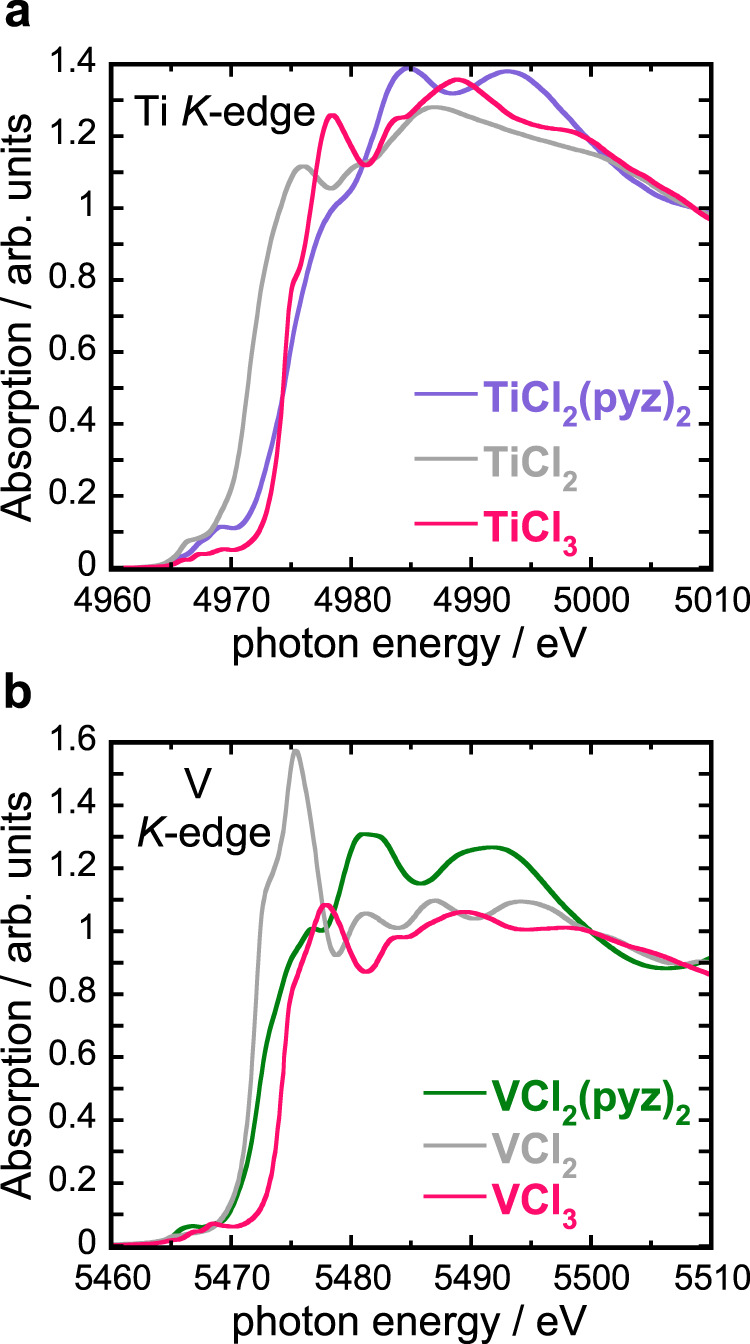
X-ray absorption spectroscopy. **a** Ti *K*-edge and **b** V *K*-edge XANES spectra of polycrystalline **TiCl**_**2**_**(pyz)**_**2**_, **VCl**_**2**_**(pyz)**_**2**_ and the reference compounds recorded at room temperature. The spectra were normalized to zero before the edge and to unity far above the edge.

To determine the oxidation state of the metal ions in **TiCl**_**2**_**(pyz)**_**2**_ and **VCl**_**2**_**(pyz)**_**2**_, X-ray absorption near edge structure (XANES) spectra were recorded at the Ti and V *K*-edges (Fig. 2). Indeed, the first inflection point of the rising edge is routinely used to assess the oxidation state of transition metal ions^33^. Here, this analysis is facilitated by the comparison with the XANES spectra recorded at the transition metal *K*-edge for M^II^Cl_2_ and M^III^Cl_3_ (M = Ti, V), which were used as references. The energy position of the *K*-edge is a function of effective metal oxidation states or, in the context of the *K*-edge XANES, a function of the effective nuclear charge (*Z*_eff_) seen by the metal core 1s electrons. As the oxidation state increases, the position of the edge shifts up in energy owing to the increase in *Z*_eff_. This effect is convincingly illustrated by the spectral changes shown in Fig. 2 and the edge positions summarized in Supplementary Table 4. In going from a M^II^ to a M^III^ formal oxidation state, the edge positions shift by ∼3 and 2.5 eV to higher energy for the titanium and vanadium chlorides, respectively. In the case of the MCl_2_(pyz)_2_ coordination solids, the energy position of the Ti *K*-edge in **TiCl**_**2**_**(pyz)**_**2**_ differs from that of the TiCl_3_ reference by 0.3 eV, whereas the difference between the V *K*-edge position in **VCl**_**2**_**(pyz)**_**2**_ and VCl_2_ is only 0.2 eV. These experimental results confirm the expected different oxidation states for the metal ions in the two MCl_2_(pyz)_2_ analogs, Ti^III^ in **TiCl**_**2**_**(pyz)**_**2**_ and V^II^ in **VCl**_**2**_**(pyz)**_**2**_. This conclusion is further supported by the XANES measurements at the Cl *K*-edge (Supplementary Information Section 4, Supplementary Figs. 7, 8 and Supplementary Table 5) and implies a chemical reduction of the pyrazine scaffold in **TiCl**_**2**_**(pyz)**_**2**_ (i.e., containing Ti^III^ and (pyz_2_)^–^) but not in **VCl**_**2**_**(pyz)**_**2**_. These results suggest the presence of a Robin-Day^34^ Class II or III mixed-valence state between the pyrazine ligands in **TiCl**_**2**_**(pyz)**_**2**_, which is commonly accompanied by an intense absorption in the near-IR region resulting from inter-valence charge transfer (Supplementary Fig. 9). However, rather than a peak, UV–vis–NIR diffuse reflectance spectroscopy reveals a broad absorption below ∼12,000 cm^–1^, that extends throughout the mid-IR. This continuum of low-lying electronic excitations is consistent with charge itineracy and a partially filled conduction band. In contrast, the **VCl**_**2**_**(pyz)**_**2**_ spectrum displays an absorption maximum at ∼12,000 cm^–1^, which may be assigned as the V^II^-localized ^4^T_2*g*_ ← ^4^A_2*g*_ (*O*_*h*_) transition^35^.

### Magnetic properties

**Fig. 3:**
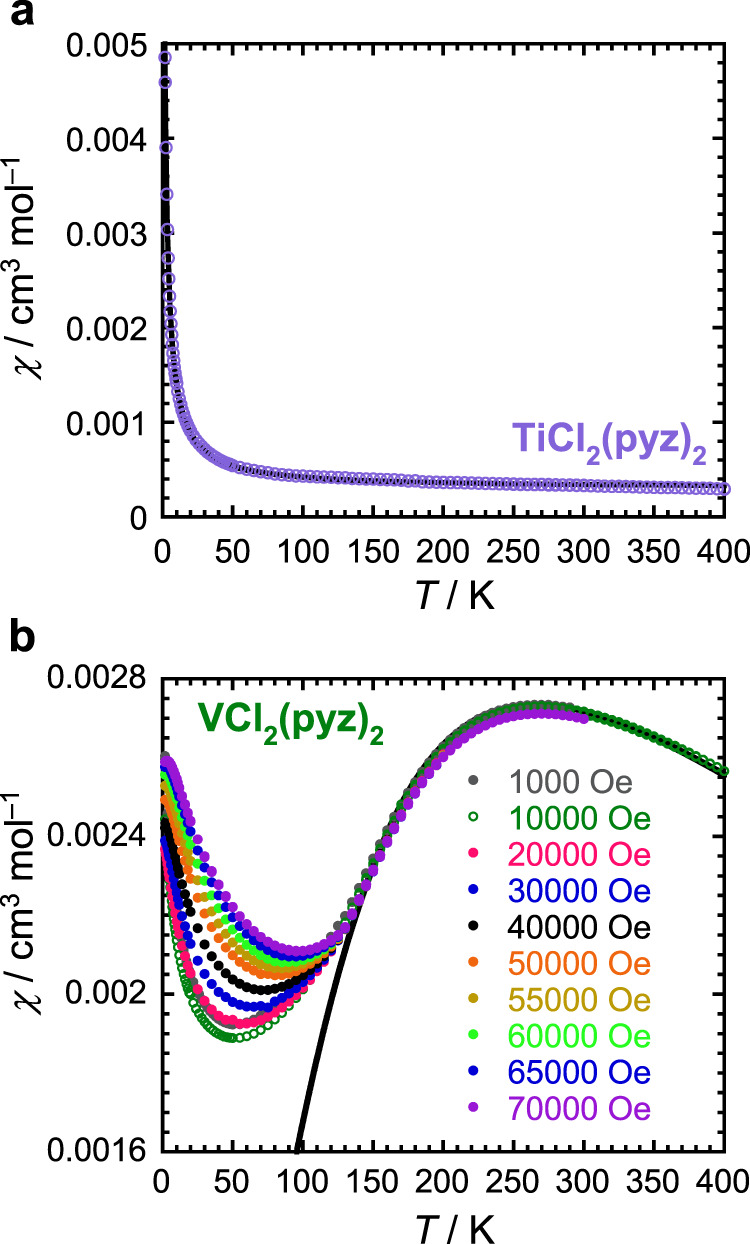
Magnetic properties. Temperature dependence of the magnetic susceptibility, *χ*, for **a**, **TiCl**_**2**_**(pyz)**_**2**_ (*μ*_0_*H* = 1.0 T) and **b**, **VCl**_**2**_**(pyz)**_**2**_ (at indicated dc fields). The solid black lines are the best fits of the experimental data to the Curie (top) and Curély (bottom) models (see main text).

In the case of **TiCl**_**2**_**(pyz)**_**2**_, the high-temperature value of the magnetic susceptibility (*χ*) - temperature product, *χT* (Supplementary Figs. 10, 11), amounts to ∼0.1 cm^3^ K mol^–1^, which is very far from the Curie constant expected for two uncoupled *S* = 1/2 centers (one Ti^III^ and one pyz^•–^ radical; 0.75 cm^3^ K mol^–1^ expected for *g* = 2). The temperature dependence of the magnetic susceptibility shown in Fig. 3 can be modeled as a simple sum of a temperature-independent term (2.9 × 10^–4^ cm^3^ mol^–1^) and a residual Curie paramagnetic contribution estimated at 1% of *S* = 1/2 impurities. This observed temperature-independent paramagnetism suggests the presence of itinerant electrons in **TiCl**_**2**_**(pyz)**_**2**_^36^.

For **VCl**_**2**_**(pyz)**_**2**_, the 400-K *χT* value of 1.03 cm^3^ K mol^–1^ is significantly lower than expected for a *S* = 3/2 V^II^ ion (1.875 cm^3^ K mol^–1^ for *g* = 2, Supplementary Fig. 10). This discrepancy is indeed anticipated in the presence of significant antiferromagnetic interactions between adjacent V^II^ spins, which are clearly evidenced by the broad maximum of the *χ vs. T* data around 250 K (Fig. 3). The magnetic susceptibility data were fitted (between 400 and 135 K) to the analytical expression derived by Curély for a square net of antiferromagnetically coupled isotropic classical spins (*S* = 3/2)^37^. The corresponding fit (black solid line in Fig. 3 and Supplementary Fig. 10) leads to *g* = 2.10(5) and *J*/*k*_B_ = –28.2(5) K (with the –2*J*∑_*ij*_*S*_*i*_*S*_*j*_ Hamiltonian definition). The strength of the superexchange coupling mediated by neutral pyrazine ligands is remarkably strong, and at least one order of magnitude larger than found in any other coordination solid pillared by neutral pyrazine^38,39^. Broken-symmetry DFT calculations performed on a dinuclear [(pyz)_3_Cl_2_V(*μ*-pyz)VCl_2_(pyz)_3_] model fragment affords a coupling constant of *J*/*k*_B_ = –79 K (Supplementary Information Sections 1 and 7). While DFT overestimates the interaction strength, the calculations reflect the unusually strong superexchange interaction in **VCl**_**2**_**(pyz)**_**2**_ mediated by pyrazine. Below ∼120 K, the susceptibility becomes strongly magnetic field-dependent (Fig. 3) and the magnetization stays far from the expected saturation value of 3 *μ*_B_ at 1.85 K and 7 T (Supplementary Fig. 12). These observations agree with the occurrence of a magnetic phase transition toward an antiferromagnetically (AF) ordered state.

Periodic lattice DFT calculations were performed on **TiCl**_**2**_**(pyz)**_**2**_ and **VCl**_**2**_**(pyz)**_**2**_ in supercells containing 92 atoms. For **VCl**_**2**_**(pyz)**_**2**_, the lowest energy spin configuration corresponds to an AF ground state with V^II^ ions and a non-magnetic ligand scaffold (Supplementary Fig. 13, Supplementary Table 6). The calculated local magnetic moments for the V center, amounting to 2.79 *μ*_B_, is close to the spin-only moment of 3 *μ*_B_ expected for V^II^. These calculations predict a large band gap of 1.5 eV, in agreement with the room-temperature transport measurements (*vide infra*). In **TiCl**_**2**_**(pyz)**_**2**_, DFT calculations confirm the Ti^III^ oxidation state, the singly reduced pyrazine scaffold, and yield a significant density of state (DOS) at the Fermi level (5 states eV^–1^ per formula unit), in agreement with the observed temperature independent paramagnetic susceptibility (Pauli paramagnetism). In addition to the Ti *d* and Cl *p*-states, C and N *p*-states of the pyrazine ligands are also found to contribute to the DOS at the Fermi level. This hybridization thus suggests a charge delocalization in the pyrazine scaffold (Supplementary Figs. 15, 16).

### Electronic conductivity, magnetoresistance, and specific heat

**Fig. 4:**
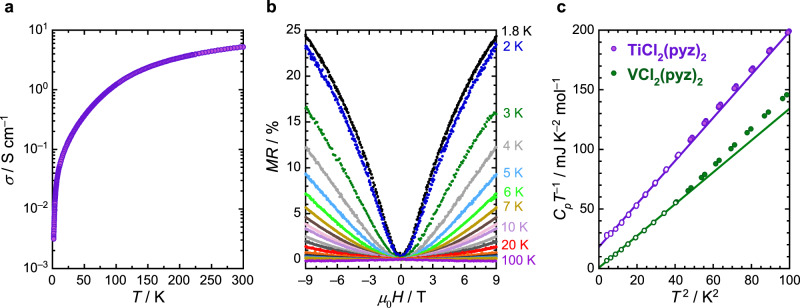
Electrical conductivity, magnetoresistance, and specific heat. **a** Temperature dependence of the pressed-pellet conductivity of **TiCl**_**2**_**(pyz)**_**2**_. **b** Magnetoresistance vs magnetic field at selected temperatures for **TiCl**_**2**_**(pyz)**_**2**_. **c** Specific heat capacity, *C*_*p*_, shown as *C*_*p*_*T*^–1^ vs *T*^2^ (all symbols) for **TiCl**_**2**_**(pyz)**_**2**_ (purple) and **VCl**_**2**_**(pyz)**_**2**_ (green). Solid lines are best fits to the data (shown in open circles) as described in the text.

The room-temperature electronic conductivity, *σ*_RT_, of **VCl**_**2**_**(pyz)**_**2**_ measured on a pressed pellet is as low as ∼10^–10^ S cm^–1^, which is a drastic reduction from the value of 0.032 S cm^–1^ observed for **CrCl**_**2**_**(pyz)**_**2**_. In contrast, *σ*_RT_ is two orders of magnitude higher for **TiCl**_**2**_**(pyz)**_**2**_ and reaches 5.3 S cm^–1^ at 300 K. Such a large *σ*_RT_ is only surpassed by a handful of coordination solids and is larger than the values found in any system with octahedrally coordinated metal-ion nodes (Supplementary Table 7)^11,25,40^. The temperature dependence of the electronic conductivity, *σ*, for **TiCl**_**2**_**(pyz)**_**2**_ is displayed in Fig. 4a. Despite the high *σ*_RT_ value and the Pauli paramagnetism evidenced in Fig. 3a, the conductivity reflects an insulating-like nature over the whole temperature range (i.e., decreasing as the temperature is lowered).

The thermal dependence of *σ* exhibits a crossover around 30 K between two different conductivity regimes, each following a *σ* = *σ*_0_ exp(–∆/*k*_B_*T*)^1/2^ law (Supplementary Fig. 17, Supplementary Information Section 8), as expected for both lightly doped semiconductors (Efros-Shklovskii model)^41^ and granular metals^18,42^. To gain more insight into the nature of the conductivity mechanism, magnetoresistance (MR) measurements were performed up to ±9 T, from 1.8 to 100 K (Fig. 4b). Strikingly, the small negative MR at high temperature (∼0.2%) evolves into a large and positive MR as the temperature is lowered, reaching 25% at 1.8 K and ±9 T. Such a strong and positive MR is generally considered to be a hallmark of metallicity. However, this increase in MR coincides with the rise of the magnetic susceptibility below ∼50 K (Fig. 3a), as demonstrated by the superposition of the experimental MR and *M*(*μ*_0_*H*)^2^ data (Supplementary Fig. 18). This observation confirms the existence of localized paramagnetic centers that are responsible for the electronic transport through spin-dependent hopping or tunneling (Supplementary Information Section 10 for further discussion).

In order to definitively conclude whether the ground state of **TiCl**_**2**_**(pyz)**_**2**_ is semiconducting or metallic, the specific heat, *C*_*p*_, was measured (Fig. 4c) and the Sommerfeld coefficient, *γ*, given by *γ* = lim_*T*→0_(*C*_*p*_*T*^–1^) was estimated experimentally. Importantly, the *γ* coefficient is directly proportional to the DOS at the Fermi level and is linked to the degree of electronic correlations (Supplementary Information Section 10 for further discussion and references). In the case of an insulator, *γ* is thus expected to vanish according to the absence of electronic states at the Fermi level. In agreement with the conductivity data (*vide supra*), the signature of an insulator is experimentally observed for **VCl**_**2**_**(pyz)**_**2**_ with *C*_*p*_*T*^–1^ extrapolating to zero at 0 K (Fig. 4c, *γ* = –0.07(35) mJ mol^–1^ K^–2^). In contrast, a residual specific heat contribution is clearly detected for **TiCl**_**2**_**(pyz)**_**2**_, yielding *γ* = 18.0(3) mJ mol^–1^ K^–2^. This Sommerfeld coefficient is an order of magnitude larger than that of a normal metal and compares well to the *γ* value of two canonical unconventional superconductors possessing strong electronic correlations: the overdoped cuprate Tl_2_Ba_2_CuO_6+*δ*_ (*γ* = 7(2) mJ mol^–1^ K^–2^)^43^ and Sr_2_RuO_4_ (*γ* = 38(2) mJ mol^–1^ K^–2^)^44^. The Sommerfeld coefficient for **TiCl**_**2**_**(pyz)**_**2**_, which is slightly larger than the value inferred from DFT calculations (*γ*_DFT_ = 9–12 mJ mol^–1^ K^–2^), is indicative of electronic correlations within Landau’s Fermi liquid theory^45^. To further confirm this conclusion, the results from the specific heat and susceptibility measurements were combined to calculate the dimensionless Wilson ratio^46^, *R*_W_ = *π*^2^*k*_B_^2^*χ*_Pauli_/(3*μ*_0_*μ*_B_^2^*γ*), which is theoretically expected to be 1 for noninteracting electrons as well as for correlated Fermi liquids. Experimentally, many strongly correlated electron systems display Wilson ratios between 1 and 2, higher values of *R*_W_ being reported for correlated Fermi liquids close to a ferromagnetic instability^47,48^. In the case of **TiCl**_**2**_**(pyz)**_**2**_, the Wilson ratio is 1.2, supporting the presence of itinerant carriers and a strongly correlated metallic ground state (Supplementary Information Section 11 for further discussion and references).

In summary, the synthesis and physical characterization of two isostructural coordination solids, **TiCl**_**2**_**(pyz)**_**2**_ and **VCl**_**2**_**(pyz)**_**2**_, have been reported in this work. Belonging to the family of layered MCl_2_(pyz)_2_ materials^27^, these new analogs display distinctively different and unique physical properties. Divalent V^II^ ions and pristine, non-reduced pyrazine ligands are found in **VCl**_**2**_**(pyz)**_**2**_, which possesses an antiferromagnetically ordered ground state below ∼120 K with unprecedently strong intra-layer V^II^–V^II^ antiferromagnetic interactions (*J*/*k*_B_ = –28 K). In contrast and like **CrCl**_**2**_**(pyz)**_**2**_^27^, **TiCl**_**2**_**(pyz)**_**2**_ contains trivalent metal-ion nodes (Ti^III^) and, formally, a singly reduced pyrazine ligand per formula unit. This peculiar electronic structure in **TiCl**_**2**_**(pyz)**_**2**_ induces not only the highest experimental electronic conductivity at room temperature (5.3 S cm^–1^) for any coordination solid based on octahedrally coordinated metal ions, but also a large positive magnetoresistance at low temperature. The combined analysis of specific heat, magnetic measurements and DFT calculations suggests the presence of a correlated Fermi liquid state in **TiCl**_**2**_**(pyz)**_**2**_. Nevertheless, the temperature dependence of the electrical resistivity, *ρ*, does not follow the expected behavior for a Fermi liquid metal (*ρ* = *ρ*_0_ + constant × *T*^2^) due to the granular nature of the material as previously observed in coordination solids. Alternatively, the Sommerfeld coefficient, which largely exceeds the typical values for non-magnetic metals, and the dimensionless Wilson ratio (close to unity) were used to firmly establish the correlated metallic ground state of **TiCl**_**2**_**(pyz)**_**2**_. The ability to chemically tune the ground state of the MCl_2_(pyz)_2_ coordination solids, from an antiferromagnetic insulator in **VCl**_**2**_**(pyz)**_**2**_, a semiconducting ferrimagnet in **CrCl**_**2**_**(pyz)**_**2**_^27^ to a strongly correlated Fermi liquid in **TiCl**_**2**_**(pyz)**_**2**_ represents a promising path towards exotic quantum phases of matter, such as superconductivity. Research on strongly correlated coordination solids remains an immensely unexplored area, but as shown in this work, the versatility of coordination chemistry combined with reducible ligands offers a powerful entry point for the exploration of new metallic and, hopefully, superconducting metal-organic materials.

## Methods

### Syntheses

All chemicals (TiCl_2_ 99.98%, VCl_2_ 85%, pyrazine ≥99%) were purchased from Sigma-Aldrich and used without further purification. Acetonitrile was dried using an Innovative Technologies solvent purification system and subsequently stored over 3 Å molecular sieves. Due to the air-sensitivity of the reagents and the final products, all manipulations were carried out under a dry dinitrogen or argon atmosphere. The reference compound, *trans*-[V^II^Cl_2_(pyridine)_4_], was synthesized through a modified literature method by the direct reaction of VCl_2_ and pyridine in a soda glass ampule at 200 °C^31^. Elemental analyses were performed at the Mikroanalytisches Laboratorium Kolbe (Oberhausen, Germany). Synthesis of **TiCl**_**2**_**(pyz)**_**2**_: A 25 mL teflon-lined stainless-steel autoclave reactor was charged with TiCl_2_ (0.2 g, 1.7 mmol) and pyrazine (2.0 g, 25 mmol), and placed in a furnace (200 °C) for 12 h. The reactor was cooled to room temperature and the black powder product of **TiCl**_**2**_**(pyz)**_**2**_ was washed with 20 mL of acetonitrile and dried in vacuo. Yield: 80–85%. Elemental analysis (calcd., found for C_8_H_8_Cl_2_N_4_Ti): C (34.5, 34.1), H (2.89, 3.12), N (20.1, 19.8), Cl (25.4, 24.8), Ti (17.2, 16.7). Synthesis of **VCl**_**2**_**(pyz)**_**2**_: **VCl**_**2**_**(pyz)**_**2**_ was synthesized following a similar procedure to that of **TiCl**_**2**_**(pyz)**_**2**_, but using VCl_2_ instead of TiCl_2_. Yield: 80-85%. Elemental analysis (calcd., found for C_8_H_8_Cl_2_N_4_V): C (34.1, 33.8), H (2.86, 2.84), N (19.9, 19.7), Cl (25.1, 25.4), V (18.1, 17.9). In order to obtain single-crystals suitable to X-ray diffraction analysis, 0.060 g of VCl_2_ (0.49 mmol) and 1.5 g of pyrazine (19 mmol) were introduced into a soda glass ampule (*V* = 21 mL) that was cooled in liq. N_2_ and flame-sealed under vacuum. The ampule was placed in a furnace, which was heated to 200 °C over 5 h. After 80 h at 200 °C, the temperature was increased to 210 °C over 30 h, maintained constant for 2 h and decreased to 200 °C over 30 h. After staying at 200 °C for 80 h, the sample returned to room temperature in about 30 h. The resulting single-crystals were isolated as described above. It is worth noting that it was not possible to obtain single-crystals of **TiCl**_**2**_**(pyz)**_**2**_ even after a similar optimization of the thermal process.

Further characterization methods, including scanning electron microscopy, crystallography, X-ray absorption and optical spectroscopies, magnetism, resistivity, specific heat and DFT calculations are described in the Supplementary Information File.

## Supplementary information

Supplementary information

Supplementary Dataset 1

Supplementary Dataset 2

Supplementary Dataset 3

Supplementary Dataset 4

Description of Additional Supplementary Files

## Data Availability

All data generated and analyzed in this study are included in the Article and its Supplementary Information, and are also available from the authors upon request. Crystallographic information has been deposited in the Cambridge Crystallographic Data Centre under the accession codes CCDC 2158352 and 2158353 (**VCl**_**2**_**(pyz)**_**2**_), 2158351 (**TiCl**_**2**_**(pyz)**_**2**_) and CCDC 2158354 (VCl_2_(py)_4_).
