# Supplementary material for: From an antiferromagnetic insulator to a strongly correlated metal in square-lattice MCl2(pyrazine)2 coordination solids
Source: Nat Commun. 2022 Sep 30;13:5766. doi: 10.1038/s41467-022-33342-5 (PMC9525593; doi:10.1038/s41467-022-33342-5)
Supplement: Supplementary file 1 — Supplementary information [file 41467_2022_33342_MOESM1_ESM.pdf]

Supplementary Information File

From an antiferromagnetic insulator to a strongly correlated metal in square-lattice $MCl_2(pyrazine)_2$ coordination solids

Panagiota Perlepe[1,2†], Itziar Oyarzabal[1,3,4,5†], Laura Voigt[6], Mariusz Kubus[6], Daniel N. Woodruff[7], Sebastian E. Reyes-Lillo[8], Michael L. Aubrey[9], Philippe Négrier[10], Mathieu Rouzières[1], Fabrice Wilhelm[11], Andrei Rogalev[11], Jeffrey B. Neaton[12,13,14], Jeffrey R. Long[9,15,16], Corine Mathonière[1], Baptiste Vignolle[2] ✉, Kasper S. Pedersen[1,6] ✉ and Rodolphe Clérac[1] ✉

[1]Univ. Bordeaux, CNRS, Centre de Recherche Paul Pascal, CRPP, UMR 5031, 33600 Pessac, France. [2]Univ. Bordeaux, CNRS, Bordeaux INP, ICMCB, UMR 5026, 33600 Pessac, France. [3]Chemistry Faculty, University of the Basque Country, UPV/EHU, 20018 Donostia-San Sebastián, Spain. [4]BCMaterials, Basque Center for Materials, Applications and Nanostructures, UPV/EHU Science Park, 48940 Leioa, Spain. [5]IKERBASQUE, Basque Foundation for Science, 48009 Bilbao, Spain. [6]Department of Chemistry, Technical University of Denmark, 2800 Kgs. Lyngby, Denmark. [7]Department of Chemistry, The University of Oxford, Oxford OX1 3QR, UK. [8]Departamento de Ciencias Físicas, Universidad Andres Bello 837-0136 Santiago, Chile. [9]Department of Chemistry, University of California Berkeley, Berkeley, CA 94720, USA. [10]Univ. Bordeaux, CNRS, Laboratoire Ondes et Matière d'Aquitaine, UMR 5798, 33400 Talence, France. [11]ESRF – The European Synchrotron, 38043 Grenoble, France. [12]Molecular Foundry, Lawrence Berkeley National Laboratory, Berkeley, Berkeley, CA 94720, USA. [13]Department of Physics, The University of California, Berkeley, Berkeley, CA 94720, USA. [14]Kavli Energy Nanosciences Institute at Berkeley, Berkeley, CA 94720, USA. [15]Department of Chemical and Biomolecular Engineering, University of California Berkeley, Berkeley, CA 94720, USA. [16]Materials Sciences Division, Lawrence Berkeley National Laboratory, Berkeley, CA 94720, USA. e-mail: baptiste.vignolle@icmcb.cnrs.fr (B.V.); kastp@kemi.dtu.dk (K.S.P.); clerac@crpp-bordeaux.cnrs.fr (R.C.). †Equally contributing authors.

e-mails: baptiste.vignolle@icmcb.cnrs.fr (B.V.); kastp@kemi.dtu.dk (K.S.P.); rodolphe.clerac@u-bordeaux.fr (R.C.).
†Equally contributing authors.

Table of Contents

1. Supplementary Methods 3

2. Supplementary Figures: Scanning electron microscopy 6

3. Supplementary Tables and Figures: Crystallographic data 8

4. Supplementary Tables and Figures: X-ray absorption spectroscopy 15

5. Supplementary Figures: Additional spectroscopic measurements 19

6. Supplementary Figures: Magnetic properties 20

7. Supplementary Discussion: Density functional theory calculations 23

8. Supplementary Discussion: Zero-field temperature dependence of the electronic conductivity of $TiCl_2(pyz)_2$ 28

9. Supplementary Discussion: Scaling of experimental magnetoresistance (MR) and magnetization in $TiCl_2(pyz)_2$ 32

10. Supplementary Discussion: Low-temperature specific heat measurements and Sommerfeld coefficient (γ) 34

11. Supplementary Discussion: Wilson ratio 36

12. Supplementary References 37

1. Supplementary Methods

Scanning electron microscopy (SEM) images were acquired on a FEI Quanta 200 ESEM FEG instrument with a 12-15 kV operating voltage range. Images were collected on microcrystalline solids deposited on carbon tapes with and without gold coating.

Crystallography. Synchrotron powder X-ray diffraction (PXRD) measurements on **$TiCl_2(pyz)_2$** and **$VCl_2(pyz)_2$** were performed at beamline I11 at the Diamond Light Source Ltd (Supplementary Figs. 3 and 4, Supplementary Table 1). Samples were loaded into 0.5 mm diameter borosilicate glass capillaries and sealed with wax. Diffraction patterns were collected at room temperature using Si-calibrated X-rays with a 0.82626(1) Å wavelength and the Mythen position-sensitive detector. It is worth mentionning that the amorphous background in Supplementary Fig. 3 originates from the borosilicate glass capillary. Williamson-Hall analysis of the diffraction peaks (with 2θ between 7.5 and 14°) for **$VCl_2(pyz)_2$** and **$TiCl_2(pyz)_2$** provided crystallite sizes of 1.5 and 0.2 μm, respectively. The structures were solved using the previously reported $NiCl_2(pyz)_2$ as a structural model input[1] for a subsequent full Rietveld refinement performed in the HighScore Plus 5.1.0 programme suite.[2] All atomic positions and thermal ellipsoids were allowed to refine freely. Occupancies of all atoms were fixed at 1 for M (M = Ti, V), Cl and N, and 0.5 for C and H in agreement with the chemical formula. Single crystal X-ray diffraction data for **$VCl_2(pyz)_2$** and **$VCl_2(pyridine)_4$** were collected on a SuperNova Dual Source CCD-diffractometer equipped with a Mo X-ray source and an Oxford Cryosystems cooler, and a Bruker D8 VENTURE diffractometer equipped with Mo Kα high brilliance IμS radiation, a multilayer X-ray mirror, a PHOTON 100 CMOS detector, and an Oxford Cryosystems low temperature device, respectively (Supplementary Tables 2 and 3, Supplementary Figs. 5 and 6). The structures were solved using the olex2.solve structure solution program[3] implemented in Olex2[4] and refined with the SHELXL refinement package using least squares minimization.[5] Crystallographic data can be obtained free of charge from The Cambridge Crystallographic Data Centre under accession codes CCDC 2158351-2158354 via https://www.ccdc.cam.ac.uk/structures/.

X-ray absorption spectroscopy. X-ray absorption near edge structure (XANES) spectra were recorded at the ID12 beamline of the European Synchrotron Radiation Facility (ESRF). For the experiments at the Ti and V *K*-edges, we used the fundamental harmonic of an Apple-II type undulator, whereas Cl *K*-edge spectra were collected using the fundamental harmonic of the Helios-II type undulator. All XANES spectra were recorded at room temperature using total fluorescence yield detection mode. The X-ray photon energy scale was calibrated using the first peak of the XANES spectra of a titanium foil assigned to 4965.6 eV. In a typical data analysis for a total fluorescence yield detected XANES spectrum, the pre-

edge background subtraction is performed using a first-order polynomial function and the edge jump is then normalized to unity. The true absorption spectrum could be obtained from the normalized fluorescence detected using standard procedures, which consider the various background contributions (fluorescence of outer electronic subshells and matrix), the angle of the X-ray beam incidence, and the solid angle of the detector.[6] The first inflection point of the rising edge of the XANES spectra is commonly used as the energy position of the absorption edge. The energy position of the transition metal *K*-edge has shown to be an attractive means to assess their oxidation state (Supplementary Table 4).[7] The Cl *K*-edge XANES spectra shown for $\mathbf{CrCl_2(pyz)_2}$, $\mathbf{TiCl_2(pyz)_2}$ and $\mathbf{VCl_2(pyz)_2}$ in Supplementary Fig. 7 exhibit characteristic double peak pre-edge structures, which are assigned to Cl 1s $\longrightarrow$ ψ^* transitions, where ψ^* is the antibonding molecular orbital obtained from linear combination of the metal *d*-orbitals and filled Cl 3*p*-orbitals of appropriate symmetry. Notably, the integrated intensity of the pre-edge features directly measures the Cl 3*p*–metal 3*d* covalency,[8,9] and as such, reflects the metal oxidation state.[10] The decrease in oxidation states leads to a decrease in covalency of the metal–Cl bond, which is reflected by less intense pre-edge structures at the Cl *K*-edge. The integrated areas of the pre-edge peaks were summed to obtain the experimental pre-edge intensities reported in Supplementary Table 5 (Supplementary Fig. 8). Since the local environment of the chlorine atoms is very similar in all three compounds, a direct comparison is then possible, and it confirms that Ti and Cr possess a higher oxidation state than V in this series of $MCl_2(pyz)_2$ materials.

Optical spectroscopy. Diffuse reflectance UV-vis-NIR spectra (Supplementary Fig. 9) were collected on a CARY 5000 spectrophotometer equipped with a Praying Mantis diffuse reflection accessory (Harrick Scientific Products, Inc.) and interfaced with Varian Win UV software. FTIR spectra (Supplementary Fig. 9) were obtained on a Bruker VERTEX 70v.

Magnetization measurements. The magnetization measurements were performed on a Quantum Design MPMS-XL SQUID magnetometer operating between 1.8 and 400 K and applied dc fields of up to 7 T. The measurements were performed on powder or microcrystalline samples (15–30 mg) sealed in polypropylene bags (size ~3 × 0.5 × 0.02 cm^3) under argon. The data were corrected for the intrinsic diamagnetic contributions of the sample and the sample holder.

Resistivity measurements. The four-point probe resistivity measurements were performed using a home-made insert installed inside a Quantum Design Physical Property Measurement System (PPMS-9) in the 1.8-400 K temperature range and applied external fields ranging from 0 to 9 T. Depending on the resistivity range, data were collected by using a Quantum Design resistivity module, an ac transport module or an external device (Keithley 2401 SourceMeter). The measurements were carried out on pellets (~7 mm diameter and 0.9-1 mm thickness, about 50-60 mg) prepared under argon with a compact

hydraulic press, applying 2 tons for 1 hour. The reported experimental data were corrected for the cell design and the pellet shape.

Specific heat. Heat capacity measurements were carried out using pressed pellets (13.0 mg for **$VCl_2(pyz)_2$** and 4.6 mg for **$TiCl_2(pyz)_2$**) with a Quantum Design Physical Property Measurement System (PPMS-9) in the 298–2 K temperature range in the absence of a dc magnetic field. A background measurement including a small amount of Apiezon-N grease (0.7 mg) used for thermal contact was measured prior to sample mounting and subtracted from the measured total heat capacity.

Density functional theory calculations. Periodic lattice spin-polarized density functional theory (DFT) calculations were performed using the Vienna Ab-initio Simulation Package (VASP).[11,12] The generalized gradient approximation of Perdew, Burke, Ernzerhof revised for solids (PBEsol)[13] plus the Hubbard U method[14] were used to describe the localization of the Ti and V *d*-orbitals. The U value chosen for V, U_V = 5.24 eV, was obtained from DFT calculations for metals in fluorine environments.[15] For Ti, U values of 0, 2, 4 and 6 eV have been used. Our calculations used a plane-wave energy cut-off of 500 eV, Monkhorst-Pack *k*-point grids, and projected augmented pseudo-potentials with 10, 5, 7, 4, 5 and 1 valence electrons for Ti, V, Cl, C, N, and H, respectively, from the VASP library.[16] Structural relaxations were performed using the PBEsol+U functional until atomic forces were smaller than 0.01 eV Å^{-1}. Projected density of states (pDOS) were computed for **$TiCl_2(pyz)_2$** and **$VCl_2(pyz)_2$**. In the case of **$VCl_2(pyz)_2$**, ferromagnetic and antiferromagnetic spin orders were considered, while we performed non-magnetic calculations for **$TiCl_2(pyz)_2$**. Magnetic moments (in units of μ_B) are computed by integrating the local spin densities on spheres around the atoms with Wigner-Seitz radii given by 1.323, 1.111, 0.863, 0.741, 0.370 Å for V, Cl, C, N and H, respectively, as implemented in VASP. Cluster DFT calculations were performed using the ORCA programme suite,[17] employing scalar relativistic effects through the zeroth-order regular approximation (ZORA).[18,19] The experimentally determined atomic coordinates of **$VCl_2(pyz)_2$** were used as input without any geometry optimization to construct a hypothetical dinuclear $VCl_2(pyz)_3$-(μ-pyz)-$VCl_2(pyz)_3$ fragment. The calculations on this model unit of **$VCl_2(pyz)_2$** employed the TPSSh functional[20] combined with the scalar-relativistically recontracted (SARC) version of the triple-ζ def2-TZVP(-f) basis set[21,22] together with the corresponding auxiliary basis. For the dinuclear fragments, a (HS) S_{total} = 3 calculation and a S_1 = 3/2 - S_2 = 3/2 broken symmetry calculation (BS(3,3)) were performed. The energy difference between the HS and BS states facilitates estimation of the exchange coupling constant, J, as defined by Yamaguchi.[23,24] The spin density plot of Supplementary Fig. 14 was rendered using the VMD[25,26] program.

2. Supplementary Figures: Scanning electron microscopy

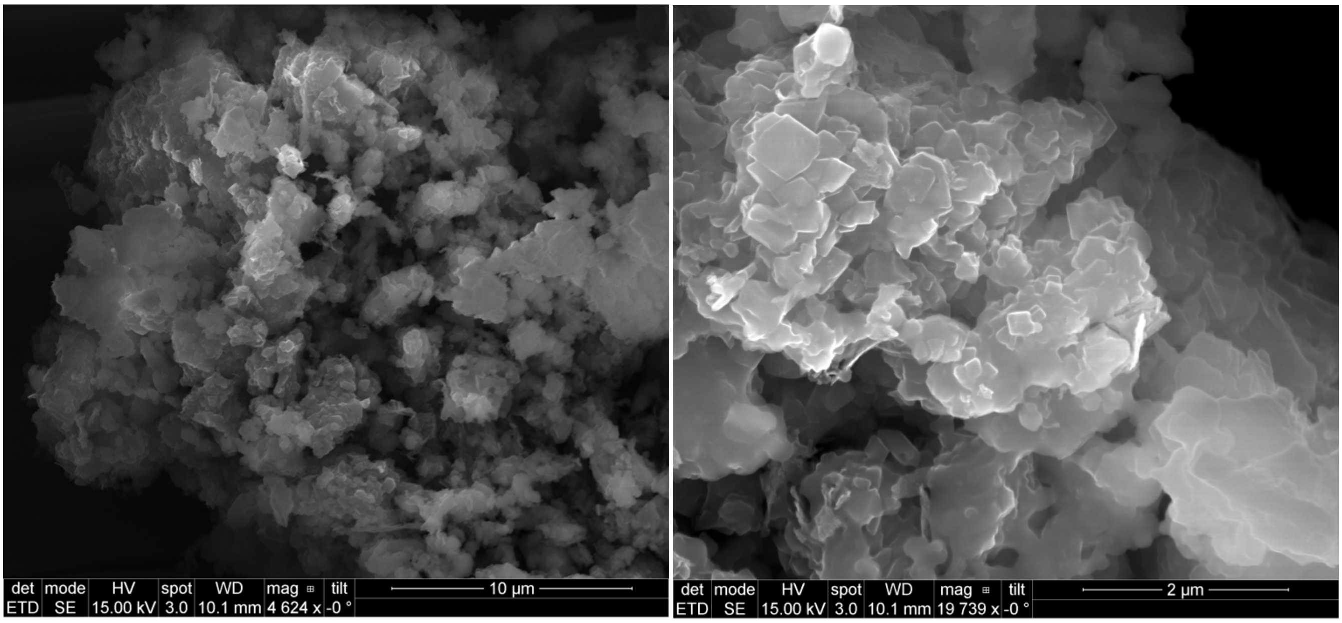

Supplementary Fig. 1. Representative SEM images of as-synthesized **$TiCl_2(pyz)_2$** at two different magnifications.

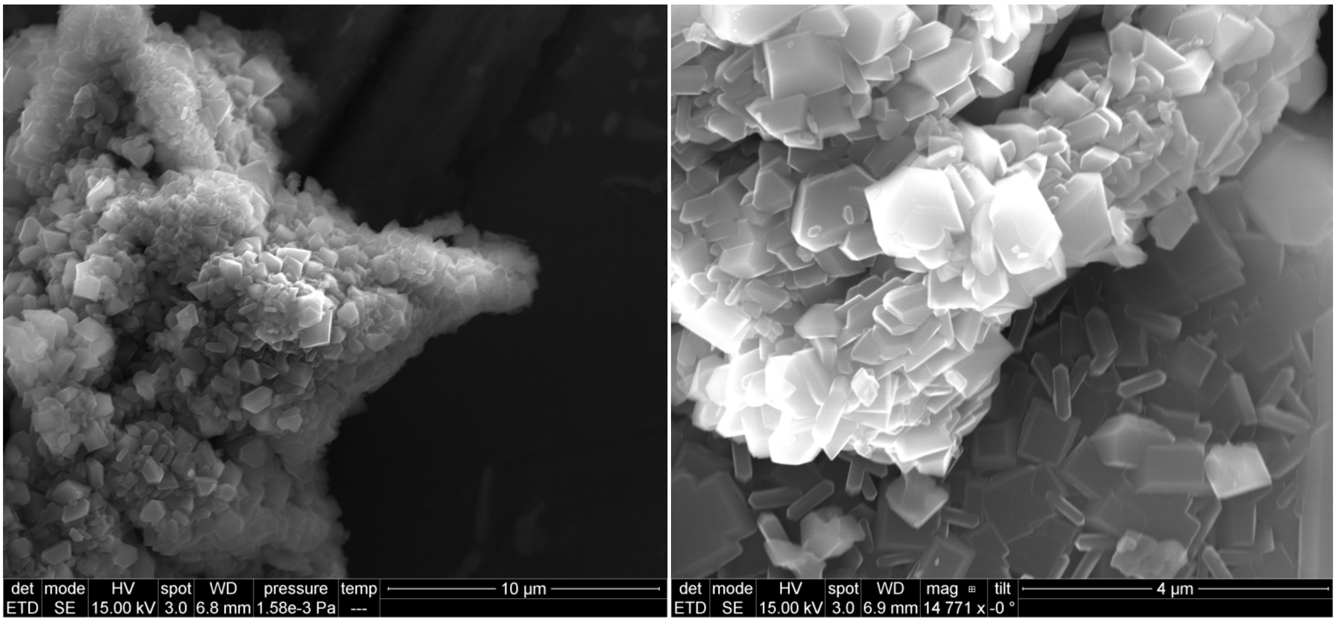

Supplementary Fig. 2. Representative SEM images of as-synthesized **$VCl_2(pyz)_2$** at two different magnifications.

3. Supplementary Tables and Figures: Crystallographic data

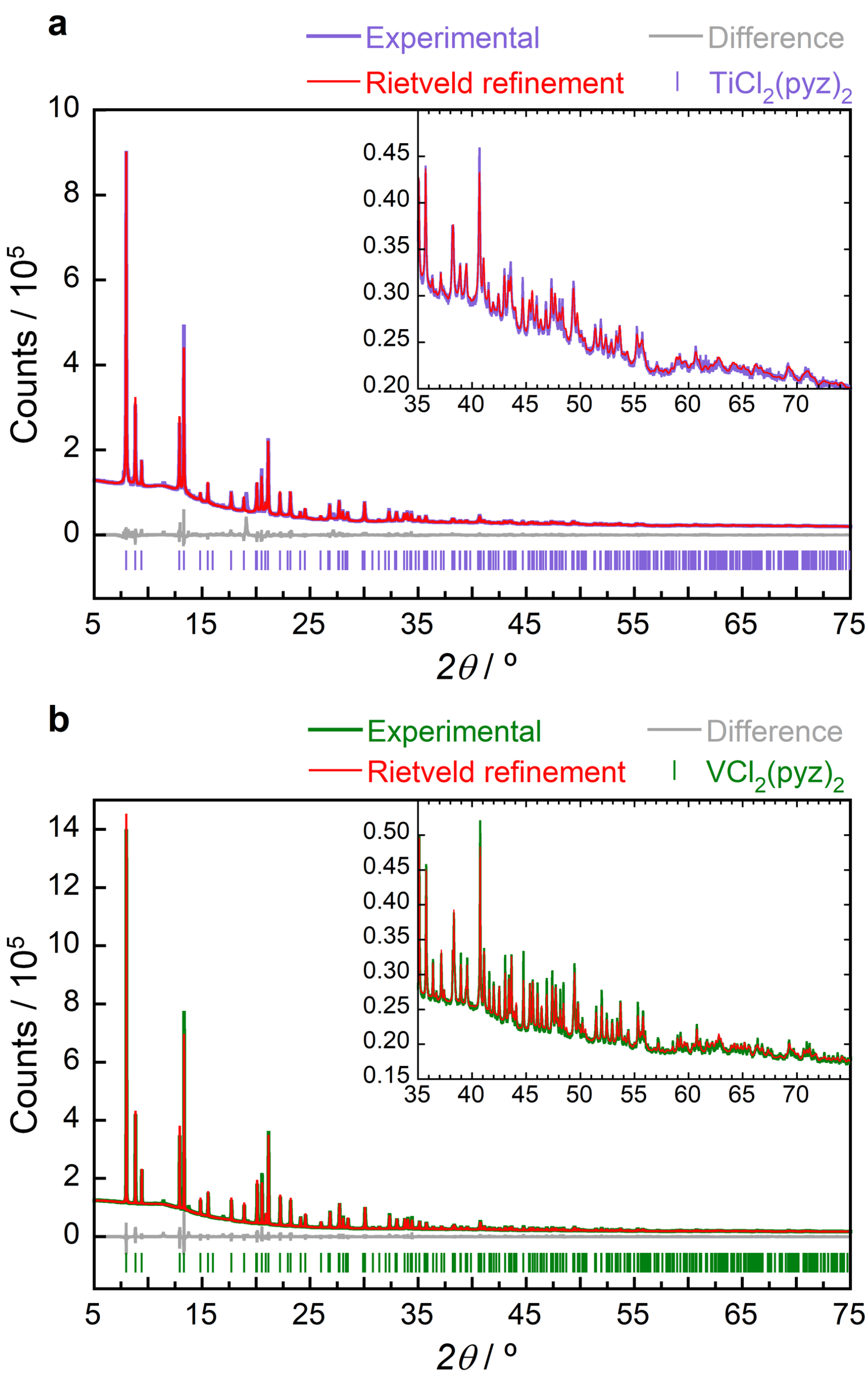

Supplementary Fig. 3. Synchrotron powder X-ray diffractograms of (top) **$TiCl_2(pyz)_2$** and (bottom) **$VCl_2(pyz)_2$** collected at room temperature with a wavelength of 0.82626(1) Å. The Rietveld refinement (red line) is shown together with the experimental difference (grey line) and the position of the Bragg peaks (purple and green bars for **$TiCl_2(pyz)_2$** and **$VCl_2(pyz)_2$**, respectively).

Supplementary Table 1. Crystallographic and refinement parameters for the synchrotron powder X-ray structure of $\mathbf{TiCl_2(pyz)_2}$ and $\mathbf{VCl_2(pyz)_2}$ (shown in Supplementary Fig. 3).

	$TiCl_2(pyz)_2$	$VCl_2(pyz)_2$
CCDC number	2158351	2158352
Empirical formula	$C_8H_8N_4Cl_2Ti$	$C_8H_8N_4Cl_2V$
Formula weight / g mol^{-1}	278.95	282.02
Temperature / K	300	300
Crystal system	Tetragonal	Tetragonal
Space group	*I*4/*mmm*	*I*4/*mmm*
a / Å	7.131(4) Å	7.1212(8)
b / Å	7.131(4) Å	7.1212(8)
c / Å	10.746(6) Å	10.737(1)
α / °	90	90
β / °	90	90
γ / °	90	90
Volume / Å^3	546.49(6)	544.51(2)
Z	2	2
Radiation, λ / Å	0.82626(1)	0.82626(1)
Goodness-of-fit on F^2	6.99	5.42
R_{wp}	0.032	0.027

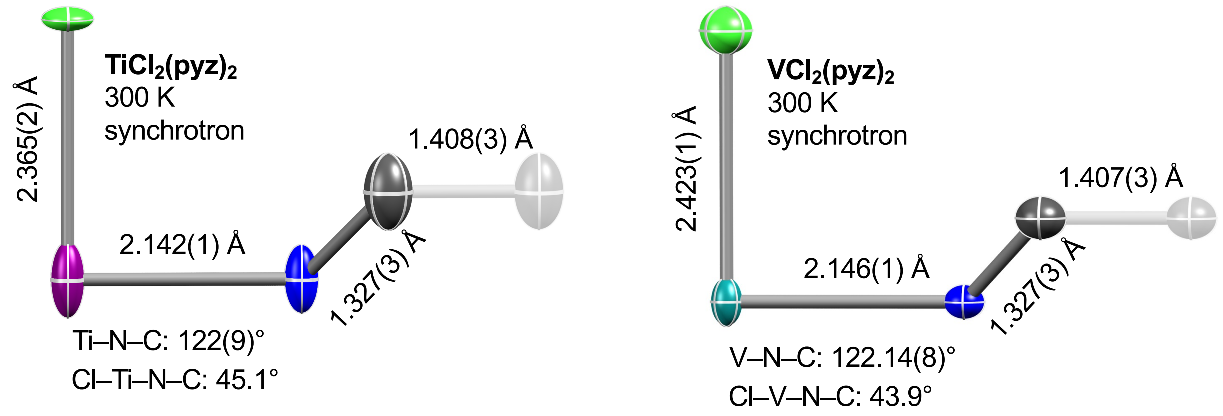

Supplementary Fig. 4. Asymmetric units of **$TiCl_2(pyz)_2$** (left) and **$VCl_2(pyz)_2$** (right) shown along the crystallographic *a* direction for powder X-ray diffraction-derived structures (at 300 K, with thermal ellipsoids at the 50% probability level). Colour code: Ti, purple; V, turquoise; Cl, green; N, blue; C, grey; H, white. The hydrogen atoms are shown as spheres with a fixed diameter.

Supplementary Table 2. Crystallographic and refinement parameters for the single-crystal X-ray structure of **$VCl_2(pyz)_2$**.

CCDC number	2158353
Empirical formula	$C_8H_8N_4Cl_2V$
Formula weight / g mol^{-1}	282.03
Temperature / K	120
Crystal system	Tetragonal
Space group	*I*4/*mmm*
a / Å	7.0899(8)
b / Å	7.0899(8)
c / Å	10.670(2)
α / °	90
β / °	90
γ / °	90
Volume / Å^3	536.4(1)
Z	2
ρ_{calc} / g cm^{-3}	1.746
μ / mm^{-1}	1.39
F(000)	283.28
Crystal size / mm^3	0.1 × 0.1 × 0.1
Radiation	Mo Kα (λ = 0.71073 Å)
θ range for data collection / °	4.1 to 28.3
Index ranges	$-8 \leq h \leq 9$
	$-9 \leq k \leq 8$
	$-14 \leq l \leq 9$
Reflections collected	1160
Independent reflections	227 [R_{int} = 0.058]
Data/restraints/parameters	227/0/23
Goodness-of-fit on F^2	1.02
Final R_1 index [$F^2 \geq 2\sigma(F^2)$]	0.031
Final wR_2 index [F^2]	0.057
Largest diff. peak/hole / e Å^{-3}	0.71/–0.87

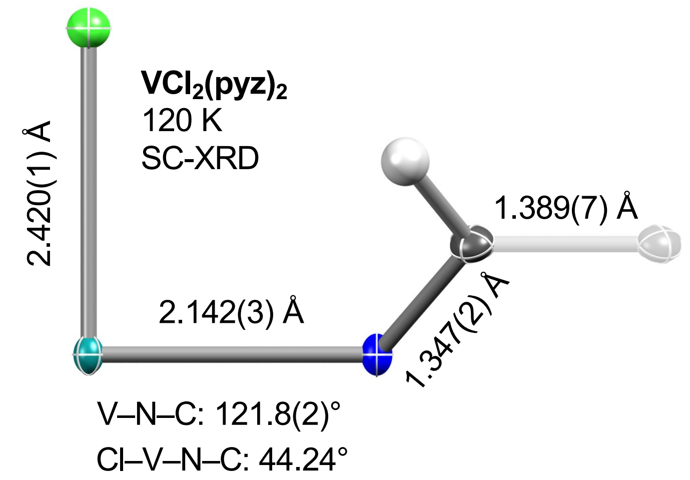

Supplementary Fig. 5. Asymmetric unit of the single crystal structure (*T* = 120 K) of **$VCl_2(pyz)_2$** shown along the crystallographic *a* direction. The thermal ellipsoids are plotted at 70% probability level and the hydrogen atom is shown as a sphere with a fixed diameter. Colour code: V, turquoise; Cl, green; N, blue; C, grey; H, white.

Supplementary Table 3. Crystallographic and refinement parameters for the single-crystal X-ray structure of **VCl_2(pyridine)$_4$**.

CCDC number	2158354
Empirical formula	$C_{20}H_{20}Cl_2N_4V$
Formula weight / g mol^{-1}	438.24
Temperature / K	120(1)
Crystal system	Tetragonal
Space group	$I4_1/acd$
a / Å	15.8193(9)
b / Å	15.8193(9)
c / Å	16.8899(8)
α / °	90
β / °	90
γ / °	90
Volume / Å^3	4226.7 (5)
Z	8
ρ_{calc} / g cm^{-3}	1.377
μ / mm^{-1}	0.73
F(000)	1800
Crystal size/mm^3	0.72 × 0.72 × 0.55
Radiation	Mo Kα (λ= 0.71073 Å)
θ range for data collection / °	3.0–25.7
Index ranges	$-18 \leq h \leq 18$
	$-19 \leq k \leq 18$
	$-20 \leq l \leq 19$
Reflections collected	15050
Independent reflections	1007 [R_{int} = 0.069]
Data/restraints/parameters	1007/63/0
Goodness-of-fit on F^2	0.98
Final R_1 index [$F^2 \geq 2\sigma(F^2)$]	0.095
Final wR_2 index [F^2]	0.205
Largest diff. peak/hole / e Å^{-3}	0.42/–0.43

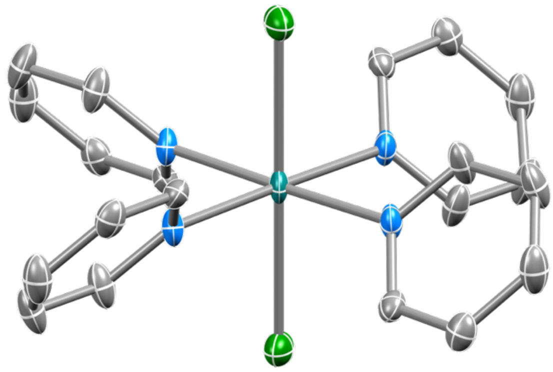

Supplementary Fig. 6. Perspective view of the structure of **VCl_2(pyridine)$_4$**. The thermal ellipsoids are plotted at 30% probability level and the hydrogen atoms are omitted for clarity. Colour code: V, turquoise; Cl, green; N, blue; C, grey.

4. Supplementary Tables and Figures: X-ray absorption spectroscopy

Supplementary Table 4. Photon energies of the Ti and V *K*-edges in the XANES spectra of **$TiCl_2(pyz)_2$**, **$VCl_2(pyz)_2$** and the reference compounds.

Compound	Ti *K*-edge energy (eV)	Compound	V *K*-edge energy (eV)
$TiCl_2(pyz)_2$	4974.7	$VCl_2(pyz)_2$	5472.3
$TiCl_2$	4971.5	VCl_2	5472.1
$TiCl_3$	4974.4	VCl_3	5474.4

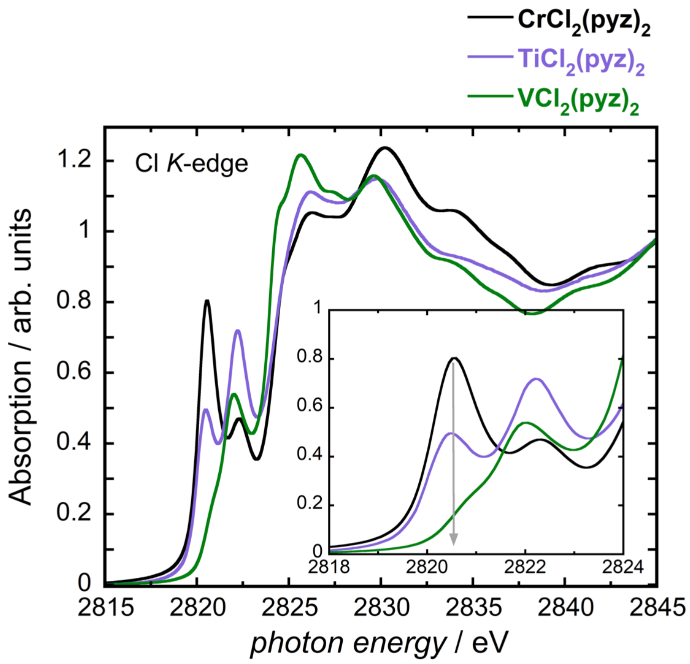

Supplementary Fig. 7. XANES spectra at the Cl *K*-edge for **$CrCl_2(pyz)_2$** (black line), **$TiCl_2(pyz)_2$** (purple line) and **$VCl_2(pyz)_2$** (green line) recorded at room-temperature. Inset: pre-edge region of the Cl *K*-edge XANES spectra for the three compounds.

Supplementary Table 5. Pre-edge features of the Cl *K*-edge XANES spectra at room-temperature for **$TiCl_2(pyz)_2$**, **$VCl_2(pyz)_2$** and the reference compounds.

	Energy position of the 1st peak (eV)	FWHM of the 1st peak (eV)	Integrated intensity of the 1st peak	Energy position of the 2nd peak (eV)	FWHM of the 2nd peak (eV)	Integrated intensity of the 2nd peak	Total intensity
$CrCl_2(pyz)_2$	2820.5	1.17	0.873	2822.1	1.33	0.384	1.257
$TiCl_2(pyz)_2$	2820.5	1.18	0.495	2822.1	1.23	0.598	1.093
$VCl_2(pyz)_2$	2820.8	0.99	0.122	2821.9	1.23	0.562	0.684

FWHM: full width at half maximum

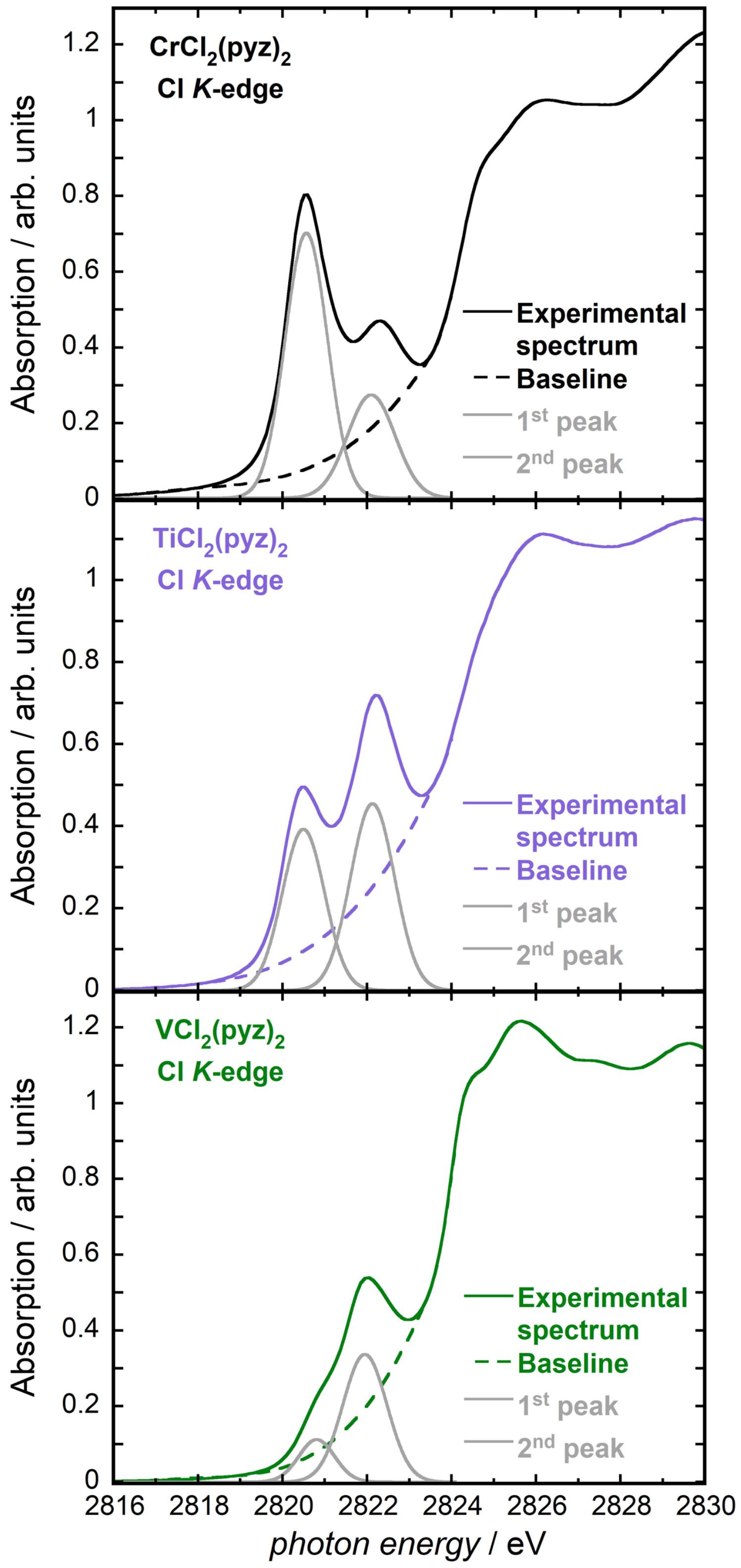

Supplementary Fig. 8. XANES spectra at the Cl *K*-edge for (top) **$CrCl_2(pyz)_2$**, (middle) **$TiCl_2(pyz)_2$** and (bottom) **$VCl_2(pyz)_2$** recorded at room-temperature and the fit of the pre-edge peaks (for further details see X-Ray Absorption Spectroscopy part in Section 1).

5. Supplementary Figures: Additional spectroscopic measurements

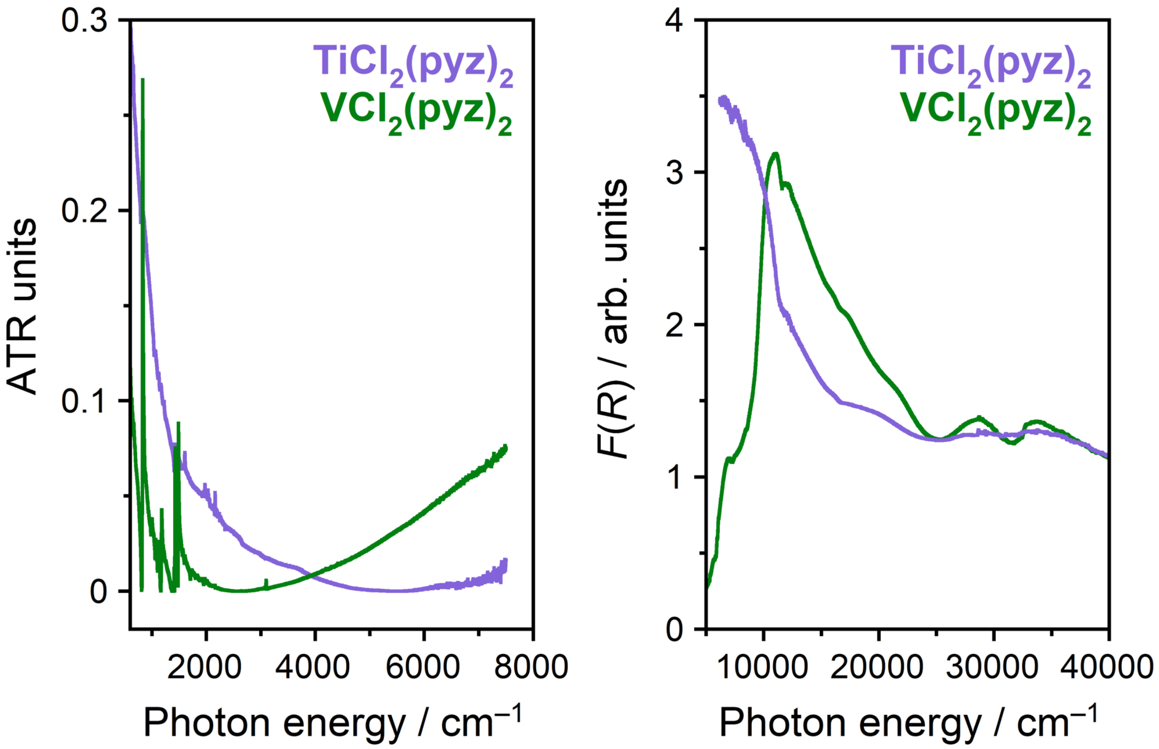

Supplementary Fig. 9. ATR-FTIR spectra (left) and diffuse reflectance UV-vis-NIR (right) spectra shown as the Kulbelka-Munk transform (*F(R)*) of **$TiCl_2(pyz)_2$** (purple) and **$VCl_2(pyz)_2$** (green).

6. Supplementary Figures: Magnetic properties

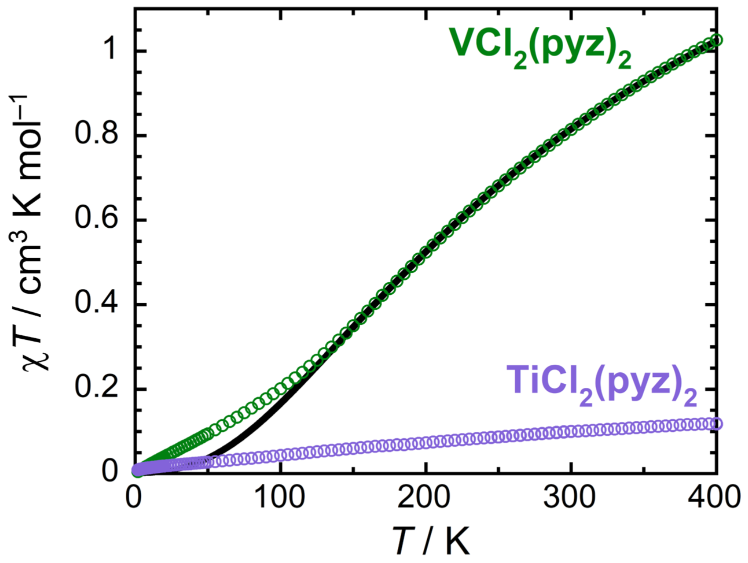

Supplementary Fig. 10. Temperature dependence of the χT product ($\mu_0 H$ = 1.0 T) of **$TiCl_2(pyz)_2$** (purple) and **$VCl_2(pyz)_2$** (green). The solid black line is the best fit of the experimental data to the Curély model as described in the main text.

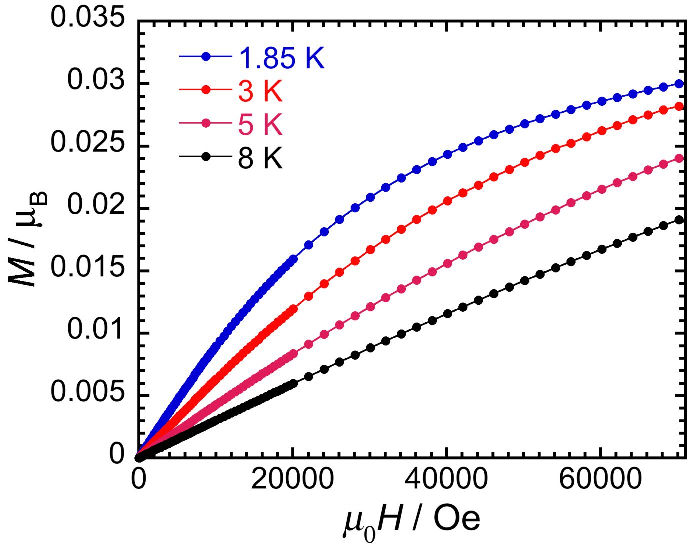

Supplementary Fig. 11. Magnetic field dependence of the magnetization for **$TiCl_2(pyz)_2$** at selected, low temperatures. Solid lines are guides for the eye.

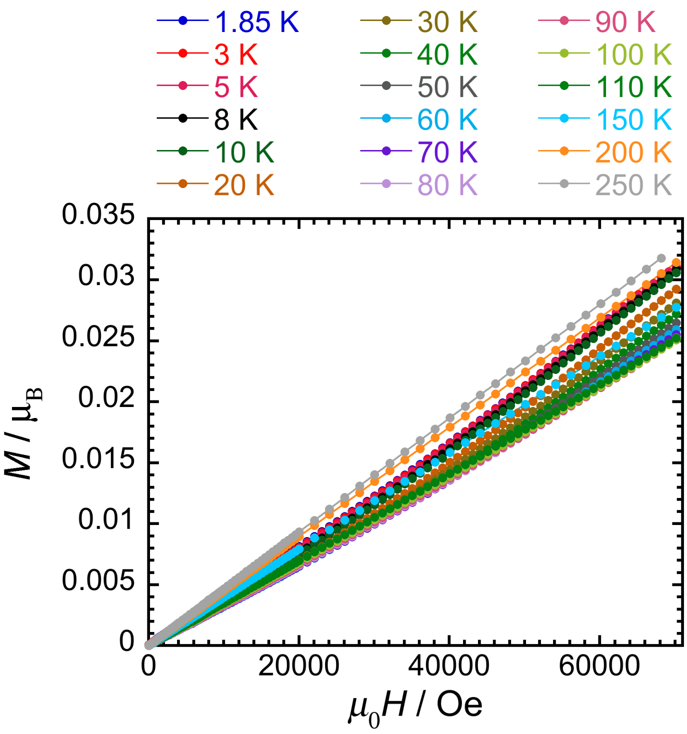

Supplementary Fig. 12. Magnetic field dependence of the magnetization for $\mathbf{VCl_2(pyz)_2}$ at selected temperatures. Solid lines are guides for the eye.

7. Supplementary Discussion: Density functional theory calculations

Supplementary Table 6. Total energy (meV/formula unit) calculations for different magnetic orders in **$VCl_2(pyz)_2$**, with respect to the corresponding ground state configuration.

Magnetic ordering (Supplementary Fig. 13)	Energy (meV/formula unit) for **$VCl_2(pyz)_2$**
FM	15.8
AF1	1314.3
AF2	924.4
AF3	0

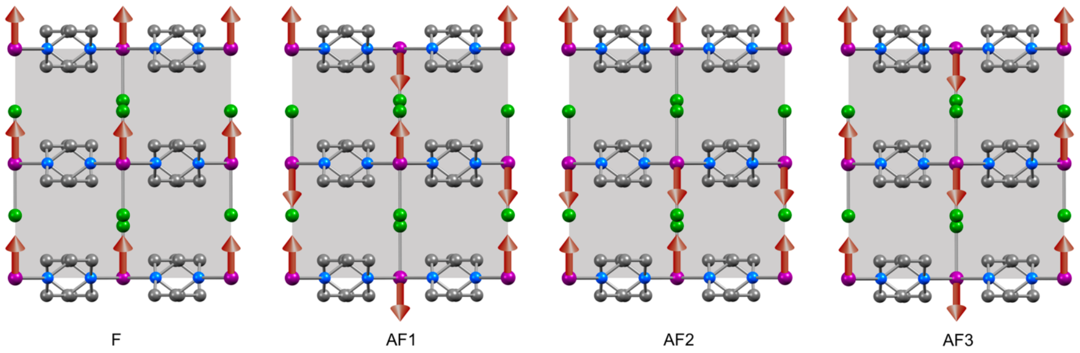

Supplementary Fig. 13. Magnetic metal atom moment orderings considered for **$VCl_2(pyz)_2$**. The structures depict the unit cell along the (110) direction.

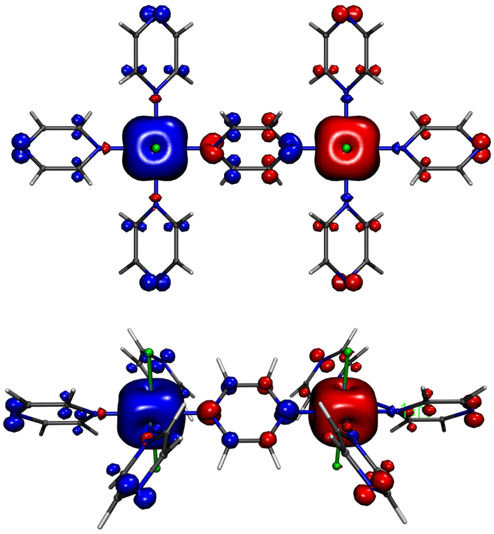

Supplementary Fig. 14. DFT-calculated spin-density in the (3,3)-broken symmetry state of the $VCl_2(pyz)_3$-(μ-pyz)-$VCl_2(pyz)_3$ fragment.

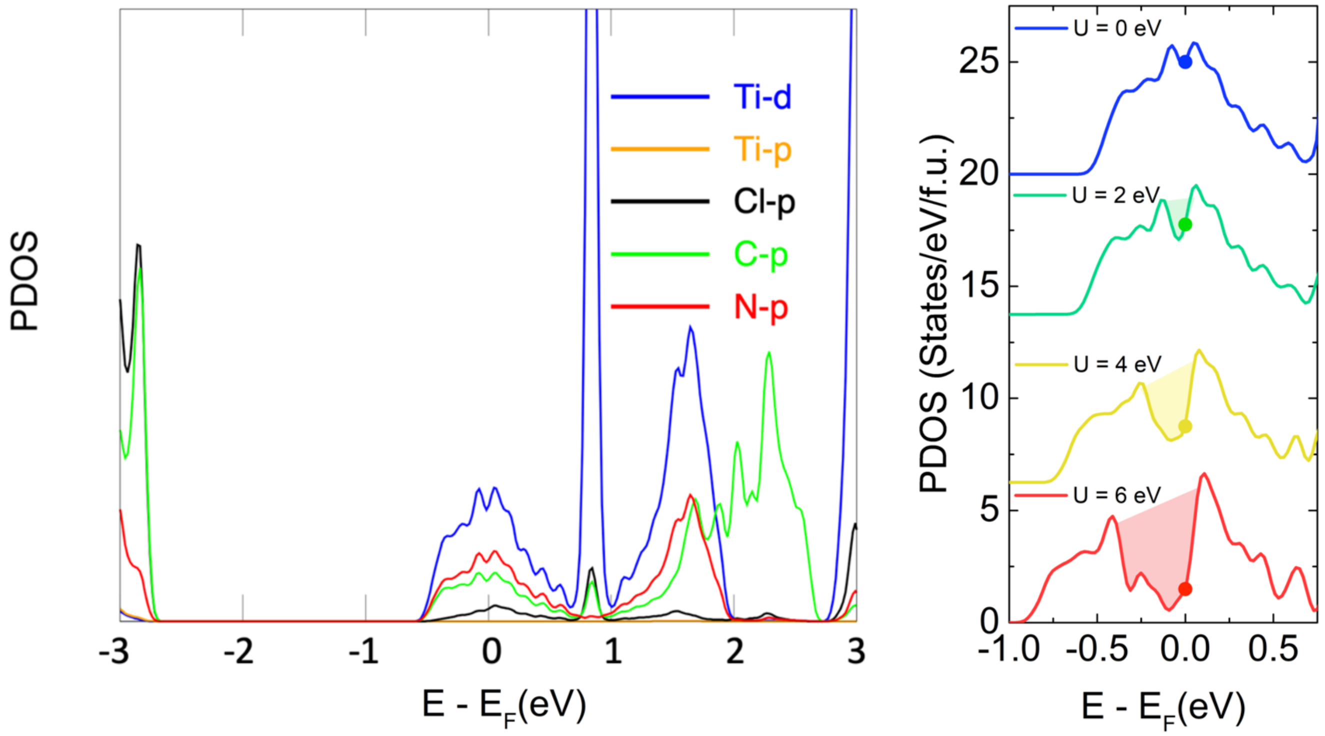

Supplementary Fig. 15. Left: Non-magnetic DFT calculation of the PDOS (Projected Density of States) of **$TiCl_2(pyz)_2$** emphasizing the contribution of each atom to the total DOS. The principal contribution comes from the *d*-electrons of the Ti ions and the *p*-electrons of C and N atoms. Right: zoom near E_F of the total PDOS from non-magnetic PBEsol + *U* calculations obtained by varying *U* from 0 to 6 eV. Solid lines represent the calculated total DOS, filled symbols the value of the DOS at E_F (5, 4, 2.5 and 1.5 states/eV/f.u. for *U* = 0, 2, 4 and 6 eV, respectively). Interestingly, a partial gap or "pseudogap" near E_F develops upon turning on the Hubbard *U* interaction (as emphasized by the shaded area). This "pseudogap" could be the signature of electronic correlations. More elaborate calculations that go beyond DFT would be required to definitively conclude on this point. Attempts to experimentally probe the DOS using ultra-violet photoelectron spectroscopy, as done for $CrCl_2(pyz)_2$,[38] were unsuccessful due to the extreme air-sensitivity of **$TiCl_2(pyz)_2$**.

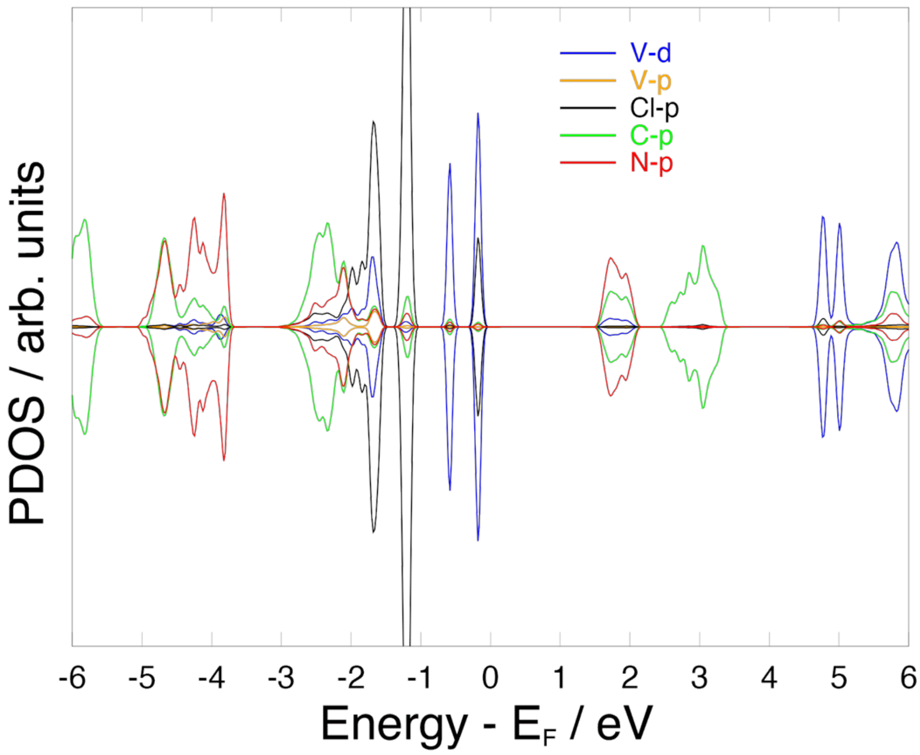

Supplementary Fig. 16. DFT calculation of the PDOS (Projected Density of States) of **$VCl_2(pyz)_2$** in the AF3 state (Supplementary Table 6) emphasizing the contribution of each atom to the total DOS.

8. Supplementary Discussion: Zero-field temperature dependence of the electronic conductivity of $TiCl_2(pyz)_2$

Despite the high value of the room-temperature electrical conductivity, σ_{RT} = 5.3 S cm^{-1}, for **$TiCl_2(pyz)_2$**, its temperature dependence can be classified as insulating-like based on the decrease of the electrical conductivity when temperature is lowered (Fig. 4a). Such a temperature dependence is typical of compounds having the Fermi level lying within the energy gap separating the valence and conduction bands. This type of systems can be classified as insulating or semiconducting depending on the magnitude of the band gap (Δ). Generally, a system is considered as semiconducting if Δ < 2 eV, and insulating if Δ > 2 eV (1 eV = 11604 K). They share a common temperature dependence of the electronic conductivity that follows an activated form: $\sigma = \sigma_0 \exp(-\Delta/k_BT)$.

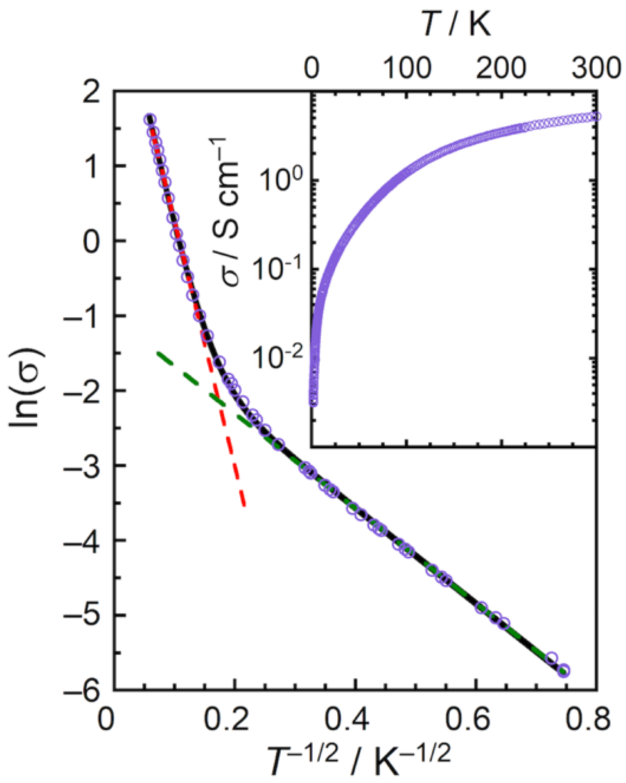

Supplementary Fig. 17. Temperature dependence of the logarithm of the electrical conductivity plotted as a function of $T^{-1/2}$, emphasizing two linear regimes. The black line represents a fit to a sum of two Efros-Shklovskii laws (individually shown in red and green dashed lines). Inset: Semi-logarithm plot of the conductivity versus temperature.

As shown clearly in Supplementary Fig. 17, the conductivity is not described by a simple activated law but instead it experiences a crossover between two regimes that rather individually follow a dependence in $\sigma = \sigma_0 \exp(-\Delta/k_BT)^{1/2}$. Three theoretical models can be invoked to analyse such a temperature

dependence. The concept of variable range hopping (VRH) was introduced by Mott[27] in 1969 to theoretically describe the low temperature electronic conduction properties of strongly disordered or amorphous semiconductors through elementary hopping processes of localized electronic states, as described by Miller and Abrahams.[28] This approach is based on the idea that an elementary hopping process between two sites depends on the energy barrier and on their distance in real space. Similar jump probabilities can be obtained from an elementary process that involves states (i) with a small energy difference that are separated by a long distance, and (ii) with a large energy separation but are close to each other in real space. In the frame of this model, the temperature dependence of the electrical conductivity can be expressed as $\sigma = \sigma_0 \exp(-T_{Mott}/T)^{1/(1+d)}$, where σ_0 is a prefactor, T_{Mott} is the gap energy scale and d is the dimensionality of the system. While using d = 1 yields the expected $\sigma = \sigma_0 \exp(-\Delta/k_BT)^{1/2}$ law, **$TiCl_2(pyz)_2$** is a two-dimensional material (Fig. 1) and thus the 1D VRH model is not adequate to describe the transport properties of this system.

Later, Efros and Shklovskii[29] refined this model to describe lightly-doped crystalline semiconductors, by incorporating a Coulomb gap into the VRH theory in order to account for electronic correlations. In crystalline semiconductors, doping is achieved by chemical substitution, which induces donor and/or acceptor impurity states. The electron associated with such a state is delocalized on a characteristic length scale called the localization length. Its wave function is most often assumed to have the form of a hydrogen-like (isotropic) wavefunction. Such an electron is weakly bound to the impurity state, which can be easily ionized. Under these conditions, if the concentration of dopant is low enough, electronic conduction is not due to free carriers but instead to charge hopping between impurity states. It can be shown that the temperature dependence of the electrical conductivity does not depend on the dimensionality of the system and can be expressed as $\sigma = \sigma_0 \exp(-T_{ES}/T)^{1/2}$, where σ_0 is a prefactor related to the localization length and T_{ES} is the temperature scale of the Coulomb gap.

Interestingly, the same expression for the temperature dependence of the electronic conductivity can be derived using a different starting point, a granular metal. This system is composed of metallic regions (called grains or granule) embedded in an insulating matrix. Electronic conduction occurs through tunnelling of electrons from one metallic grain to another. More accurately, co-tunnelling events (one in and one out of the grain), that are either elastic or inelastic, are responsible for electronic conduction. This granular metal (GM) model then considers a randomly resistive network to yield a temperature dependence similar to that of the Efros-Shklovskii (ES) model: $\sigma = \sigma_0 \exp(-T_0/T)^{1/2}$.[30] Supplementary Fig. 17 displays ln(σ) as a function of $T^{-1/2}$ to highlight the temperature domains where the electrical conductivity can be described by a ES/GM law ($\sigma = \sigma_0 \exp(-\Delta/k_BT)^{1/2}$). An experimental crossover around 30 K between two different conduction regimes can be clearly identified. This experimental temperature dependence can be reproduced using a simple parallel resistor model (leading to a sum of two ES/GM laws; black line in Supplementary Fig. 17) for which both conduction pathways act in parallel over the whole temperature range. With this approach, the characteristic parameters of the two conduction regimes can be determined: the high temperature regime possesses a large energy

gap, Δ/k_B, of 1360(11) K (σ_0 = 43(1) S cm^{-1}; red dashed line), while the low temperature one has a much smaller gap of 41(1) K (σ_0 = 0.36(1) S cm^{-1}; green dashed line).

The ES (or GM) conductivity model applies primarily to the low temperature description of electronic conduction through hopping (or tunnelling). Upon increasing temperature, the Efros-Shklovskii mechanism of hopping conductivity is expected to break down when $T > \Delta/k_B$, giving rise to nearest neighbour hopping or to thermally activated transport of free carriers, both of them being characterized by a T^{-1} dependence of ln(σ). This is clearly not what is experimentally observed in the reported measurements (Supplementary Fig. 17). For granular metal transport, there is a priori no reason either to expect two regimes of conduction.

Because of the polycrystalline nature of the pellets used for the conductivity measurements, it is not possible to conclude at this stage whether the observed charge transport is intrinsic, due to hopping between localized impurity states (semiconducting model as described by the ES theory) or extrinsic, due to tunnelling events between metallic grains (granular metal model). In order to get more insight into the fundamental mechanism at play in the electronic conduction for **$TiCl_2(pyz)_2$**, magnetoresistance measurements were performed (see Section 9).

Supplementary Table 7. Coordination solids with octahedrally coordinated metal centres and highest room temperature electrical conductivity values.

Compounds	σ (S cm^{-1}) at RT	σ method	Reference
Fe(1,2,3-triazolate)$_2$(BF$_4$)$_{0.33}$	0.3 (1)	2-probe pellet	31
Fe$_2$(BDT)$_3$	1.2(4)	2-probe sc	32
K$_{0.98}$Fe$_2$(BDP)$_3$	0.025	2-probe sc (FET)	33
[Fe$_2$(dhbq)$_3$][(Bu$_4$N)$_2$]	0.16(1)	2-probe pellet	34
[Fe$_2$(Cl$_2$dhbq)$_3$][(Me$_2$NH$_2$)$_2$]·2H$_2$O·6DMF	0.014	2-probe pellet	35
[Fe$_2$(Cl$_2$dhbq)$_3$][(Me$_2$NH$_2$)$_2$]	1.0(3)·10^{-3}	2-probe pellet	35
[Fe$_2$(Cl$_2$dhbq)$_3$][(Cp$_2$Co)$_{1.43}$(Me$_2$NH$_2$)$_{1.57}$]·4.9DMF	5.1(3)·10^{-4}	2-probe pellet	35
[Fe$_2$(Cl$_2$dhbq)$_3$][(H$_3$O)(H$_2$O)(phz)$_3$]	0.031(8)<br>1.0(9)·10^{-4}	2-probe sc (‖)<br>2-probe sc (⊥)	36
[Fe$_2$(Br$_2$dhbq)$_3$][(H$_3$O)(H$_2$O)(phz)$_3$]	3(1)·10^{-3}<br>6(2)·10^{-6}	2-probe sc (‖)<br>2-probe sc (⊥)	36
[Ti$_2$(Cl$_2$dhbq)$_3$][(Me$_2$NH$_2$)$_2$]·4.7DMF	2.7(2)·10^{-3}	2-probe pellet	37
[V$_2$(Cl$_2$dhbq)$_3$][(Me$_2$NH$_2$)$_2$]·6.4DMF	0.45(3)	2-probe pellet	37
[Cr$_2$(Cl$_2$dhbq)$_3$][(Me$_2$NH$_2$)$_2$]·4.4DMF	1.2(1)·10^{-4}	2-probe pellet	37
CrCl$_2$(pyz)$_2$	3.2·10^{-2}	2-probe pellet	38
Cd$_2$(azbpy)$_2$(HIP)$_2$	1.86	2-probe film diode	39
[Cd(NDI-py)(OH$_2$)$_4$](NO$_3$)$_x$·nDMA<br><br>[Cd(NDI-py)(OH$_2$)$_4$](NO$_3$)$_x$·mH$_2$O	(1.0 – 3.3)·10^{-3}<br>(1.5 – 7.6)·10^{-6}<br>(1.2 – 3.7)·10^{-2}	sc<br>2-probe pellet<br>2-probe pellet	40
Fe-HHTP	1.1·10^{-4}<br>5.6·10^{-3}	2-point pellet<br>4-point pellet	41

Abbreviations: BDT = benzene-1,4-ditetrazolate; BDP = benzene-1,4-dipyrazolate; H$_2$dhbq = 2,5-dihydroxy-1,4-benzoquinone; Bu$_4$N = tetrabutylammonium; H$_2$Cl$_2$dhbq = 2,5-dichloro-3,6-dihydroxo-1,4-benzoquinone; Me$_2$NH$_2$ = dimethylammonium; Cp$_2$Co = cobaltocene; phz = phenazine; H$_2$Br$_2$dhbq = 2,5-dibromo-3,6-dihydroxo-1,4-benzoquinone; pyz = pyrazine; azbpy = *trans*-4,4′-azobispyridine; HIP = 5-hydroxyisophthalate; NDI-py = *N*,*N*′-di(4-pyridyl)-1,4,5,8-naphthalenetetracarboxdiimide; HHTP = hexahydroxytriphenylene. sc = single crystals; FET = field-effect transistor measurements. ‖ and ⊥ indicate the direction of measurements along and perpendicular to the hexagonal layers.

9. Supplementary Discussion: Scaling of experimental magnetoresistance (MR) and magnetization in $TiCl_2(pyz)_2$

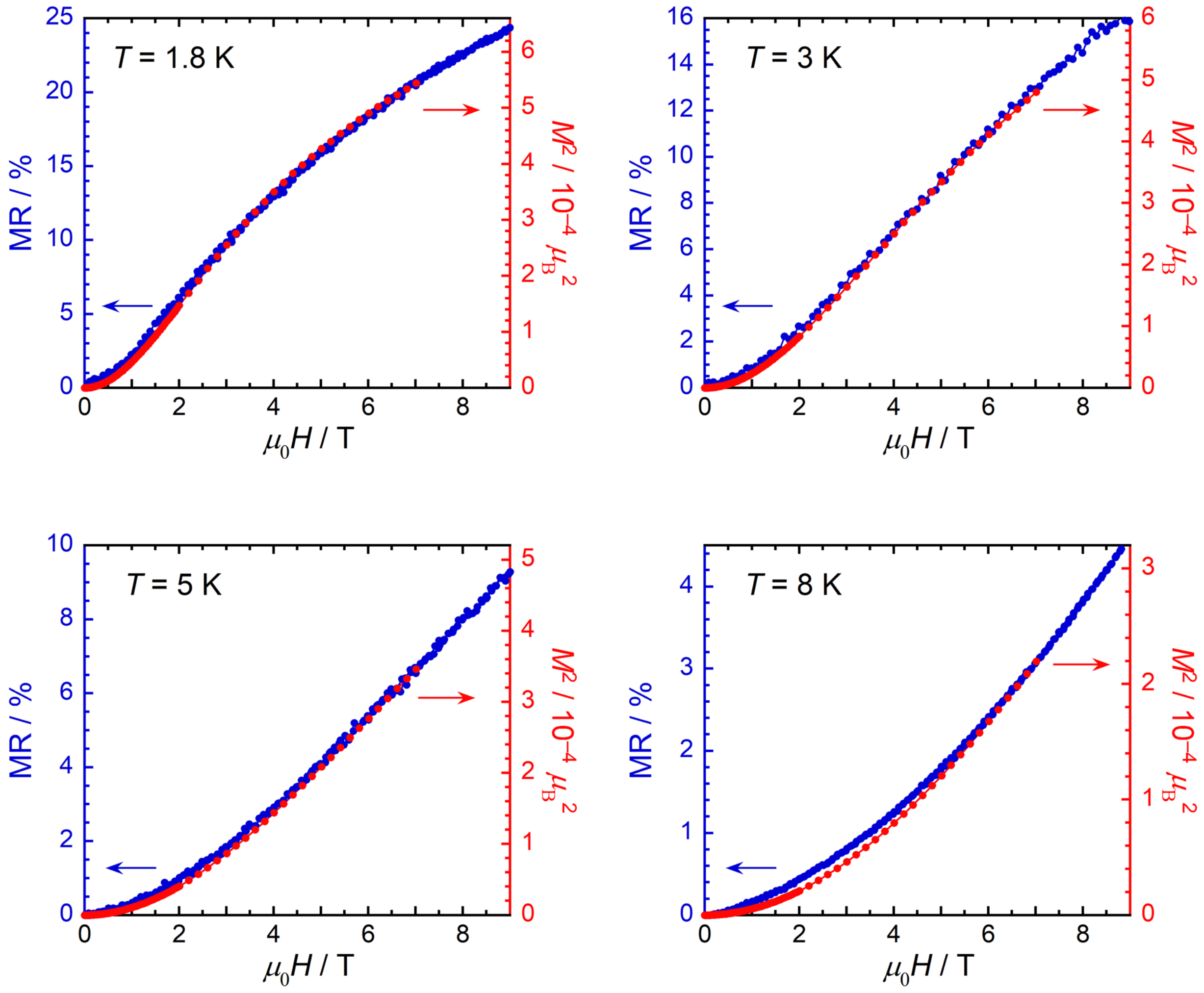

Supplementary Fig. 18. Comparison of the magnetic field dependence of the MR (left axis, blue symbols) and magnetization (M^2, right axis, red symbol) at different temperatures for $\mathbf{TiCl_2(pyz)_2}$.

The magnetoresistance data presented in Fig. 4b display rich temperature and field dependences. At the highest measured temperature (100 K), the MR is very small (0.2%) and negative. Upon lowering the temperature, the magnetoresistance changes sign and becomes positive between 100 and 80 K. Upon further lowering of the temperature, the magnetoresistance increases and develops a non-trivial field dependence, reaching values as high as 25% at 1.8 K. A strong and positive magnetoresistance at low temperature is usually the hallmark of metallic systems. For a one-band metal, the MR is quadratic in applied magnetic field, whereas it displays a more complex dependence for multi-band metals. In

order to better understand the origin of this strong MR developing at low temperature, the magnetic field dependence of the MR (left axis, blue circles) is compared in Supplementary Fig. 18 with that of the square of magnetization (M^2, right axis, red circles). Both quantities display the same field dependence. Therefore, the paramagnetic impurities measured in the magnetic measurements are clearly involved in the electrical conduction through spin dependent hopping processes (if the system is a doped semiconductor described by the ES theory) or through spin dependent tunnelling processes (if the system is a granular metal). It is remarkable to note that a doping concentration as low as 1% (based on the Curie constant; Fig. 3) is enough to dominate the electronic conduction in **$TiCl_2(pyz)_2$**. Given the polycrystalline nature of the measured samples, the above conductivity and MR analyses cannot discriminate if the ground state is best described as a lightly-doped semiconductor or as a granular metal.

10. Supplementary Discussion: Low-temperature specific heat measurements and Sommerfeld coefficient (γ)

The low temperature asymptotic behaviour of the total specific heat C_p of non-magnetically ordered systems is given by $C_p = C_e T + C_{ph} T^3$, where C_e and C_{ph} correspond to the electronic and phononic contributions to the total specific heat, respectively. The Sommerfeld coefficient, defined as $\gamma = \lim_{T\to 0}(C_p/T)$, is usually extracted by extrapolating the linear variation of C_p/T vs T^2 measured at the lowest temperatures. Fig. 4c presents such an analysis where the solid lines represent the best fits for **$TiCl_2(pyz)_2$** and **$VCl_2(pyz)_2$** yielding γ = 18.0(3) and –0.07(35) mJ mol^{-1} K^{-2} respectively. The case of γ = 0 is expected for insulators, where a large band gap is present at the Fermi level (E_F). Indeed, the Sommerfeld coefficient of a free electron gas reads $\gamma_0 = (\pi^2/3)\, k_B^2\, N(E_F)$, with $N(E_F)$ being the density of state (DOS) at the Fermi level. Therefore, γ = 0 implies that there is no DOS at the Fermi level. Within the Fermi liquid theory, Sommerfeld coefficient is further renormalized by electron-electron and electron-phonon interactions, which enter into the effective mass, m^*, of the charge carriers, leading to $\gamma = \gamma_0\, m^*$.

It is instructive to quote here some γ values reported in the literature. For non-magnetic metals with weak electron-electron interactions, γ ranges between 0.65 mJ mol^{-1} K^{-2} (0.7 mJ mol^{-1} K^{-2}) for Ag (Cu) and 1.4 mJ mol^{-1} K^{-2} for Al. The high value of γ in aluminium is considered to originate from a higher effective mass due to stronger electron-phonon interactions.[42] Quite generally, γ values well above 1 mJ mol^{-1} K^{-2} indicate a substantial degree of electronic correlations within the system. As an example, a γ value of 7(2) mJ mol^{-1} K^{-2} has been estimated for the unconventional superconductor $Tl_2Ba_2CuO_{6+\delta}$ in which strong electronic correlations are expected to be relevant.[43] For Sr_2RuO_4, another strongly correlated unconventional superconductor, γ value of 38(2) mJ mol^{-1} K^{-2} has been reported.[44] In these two systems, despite the presence of strong electronic correlations (with effective masses up to $m^* = 6\, m_0$ for $Tl_2Ba_2CuO_{6+\delta}$,[45] and $m^* = 12\, m_0$ for Sr_2RuO_4,[46] m_0 being the free electron mass), it has been demonstrated that the ground state of these systems can still be described as a correlated Fermi liquid. Even larger γ values are observed in heavy fermion materials, in part due to their strongly renormalized effective mass (that can be as high as 400 m_0). Looking now at molecular compounds such as the Bechgaard salts, the reported γ values range between 19 mJ mol^{-1} K^{-2} for κ-(BEDT-TTF)$_2$I$_3$ to 26 mJ mol^{-1} K^{-2} for α-(BEDT-TTF)$_2$KHg(SCN)$_4$.[47,48,49,50,51] These molecule-based compounds are both superconductors, for which strong electronic correlations are believed to play a key role in the occurrence of a superconducting state.

In order to get more insight into the γ value obtained for **$TiCl_2(pyz)_2$**, and quantify the degree of the electronic correlations, the γ value was also estimated from the calculated DOS presented in Supplementary Fig. 15 using $\gamma_{DFT} = (\pi^2/3)\, k_B^2\, N_{DFT}(E_F)$, with $N_{DFT}(E_F)$ being the calculated DOS. For U =

0 and 2 eV, $N_{DFT}(E_F)$ is 5 and 4 states/eV/f.u. respectively, which correspond to γ_{DFT} = 11.8 and 9.4 mJ mol^{-1} K^{-2}, respectively. These DFT values are significantly lower than the experimentally determined one, γ = 18 mJ mol^{-1} K^{-2}. The high γ value is thus due to electronic correlations, the effective mass m* being significantly larger than the band mass m_B. The presence of electronic correlations could also explain the appearance of a partial gap in the DOS at E_F as presented in Supplementary Fig. 15.

Coming back to the γ values reported in this work, a value of –0.07(35) mJ mol^{-1} K^{-2} confirms unambiguously the insulating ground state of **$VCl_2(pyz)_2$**. This result implies a substantial energy gap at the Fermi level in agreement with the electrical conductivity measurements and the DFT calculations. For **$TiCl_2(pyz)_2$**, the large γ value of 18 mJ mol^{-1} K^{-2} is comparable to those measured in correlated Fermi liquids such as the Bechgaard salts, the cuprates, and Sr_2RuO_4. While impurity states pinned at the Fermi level could in principle give rise to a finite γ value, its magnitude should be very small, γ < 1 mJ mol^{-1} K^{-2}, due of the low DOS involved. Given the small amount (~1%) of impurities inferred from magnetic susceptibility measurements (Fig. 3a), it is impossible to attribute the large γ value reported herein to such impurities. Therefore, and despite the insulating-like temperature dependence of the electrical conductivity reported for **$TiCl_2(pyz)_2$** (Fig. 4a), its intrinsic ground state must be metallic in nature. Hosting itinerant carriers with strong electronic correlations are thus likely the source of the large γ value. The experimental insulating-like behaviour of the conductivity (Fig. 4a) is then extrinsic and dominated by electronic transport across grain boundaries of the polycrystalline samples, as described in the granular metal model.

11. Supplementary Discussion: Wilson ratio

To get more insight into the ground state of **$TiCl_2(pyz)_2$**, the Wilson ratio was calculated combining experimental magnetic susceptibility data performed at 1 T with low temperature specific heat. The dimensionless Wilson ratio, R_W, that relates the Pauli susceptibility (χ_{Pauli}) to the Sommerfeld coefficient (γ) is given by $R_W = \pi^2 k_B^2 \chi_{Pauli}/(3\mu_0\mu_B^2\gamma)$, where μ_B is the Bohr magneton, μ_0 the vacuum permeability, χ_{Pauli} is expressed in m^3 mol^{-1} and γ in J mol^{-1} K^{-2}. For a free electron gas, the Pauli susceptibility is given by $\chi_{Pauli} = \chi_0 = \mu_B^2 N(E_F)$ and the Sommerfeld coefficient is $\gamma = \gamma_0 = (\pi^2/3)\, k_B^2\, N(E_F)$. It immediately follows that $R_W = 1$ for a free electron gas. Switching on the electronic interactions to describe a Fermi liquid ground state, the Pauli susceptibility and the Sommerfeld coefficient become $\chi_{Pauli} = \chi_0\, m^*/m_0$ and $\gamma = \gamma_0\, m^*/m_0$ respectively, with m^* being the effective mass of the system that incorporates both electron-electron and electron-phonon interactions and m_0 is the mass of the free electron. Thus, $R_W = 1$ is also expected in the case of a Fermi liquid, even in the presence of strong electronic correlations. It is worth noting that this result continues to hold in the case of a weakly interacting Fermi liquid, for which the effective mass $m^* = m_B$, m_B being the band mass. Experimentally, most R_W values reported for non-interacting as well as interacting Fermi liquid systems lie between 1 and 2. Theoretically, a $R_W = 2$ value has been demonstrated for Kondo systems,[52] where it is necessary to go beyond the approximation of the Pauli susceptibility given above to incorporate the first Landau parameter. R_W values larger than 2 have also been reported in materials that are near ferromagnetic instabilities such as $Sr_2Ru_3O_7$ with R_W = 10.[53]

In order to calculate the Wilson ratio for **$TiCl_2(pyz)_2$**, the Pauli susceptibility, $\chi_{Pauli} = 2.9 \times 10^{-4}$ cm^3 mol^{-1}, was estimated from the fitting of the experimental data shown in Fig. 3a. Using the experimentally determined Sommerfeld coefficient of 0.018 J mol^{-1} K^{-2}, a Wilson ratio of 1.2 is obtained in excellent agreement with the expected value for a Fermi liquid and remarkably close to that of BEDT-TTF based molecular superconductors.[54] Given that the large value of the measured Sommerfeld coefficient significantly exceeds the value theoretically predicted by DFT and that the R_W value is close to 1, the ground state of **$TiCl_2(pyz)_2$** is best described as a correlated Fermi liquid. Therefore, thermodynamic measurements demonstrate here that the insulating-like behaviour of the electrical conductivity is extrinsic and is most likely related to the granular nature of the measured samples.

To conclude, it should be noted that the Landau's Fermi liquid theory predicts that the three thermodynamic quantities, the Sommerfeld coefficient (γ), the electronic compressibility (κ), and the magnetic susceptibility (χ), are related to each other by the following relations:

$$\frac{\gamma}{\gamma_0} = \frac{m^*}{m_0}\ ;\ \frac{\kappa}{\kappa_0} = (1 + F_0^s)\frac{m^*}{m_0}\ ;\ \frac{\chi}{\chi_0} = (1 + F_0^a)\frac{m^*}{m_0}$$

where m^* is the effective mass, $F_0^{a,s}$ the first Landau parameter, m_0, γ_0, κ_0, and χ_0 are the free electron values. While the estimation of κ is beyond the present discussion, it is extremely convincing to demonstrate here that the experimental γ and χ values for **$TiCl_2(pyz)_2$** follow perfectly the thermodynamic relations expected within the Landau's Fermi liquid framework. Therefore, the presence of a Fermi liquid metallic state is confirmed in **$TiCl_2(pyz)_2$** despite the experimental absence of its most direct signature: an electrical resistivity that grows as T^2 at low temperature. While one should always keep in mind that the resistivity is not a thermodynamic quantity, the insulating-like temperature dependence of the electrical conductivity (Fig. 4a) is extrinsic to the **$TiCl_2(pyz)_2$** material and induced by the polycrystalline (granular) nature of the measured sample.

12. Supplementary References

1. Liu, J., Goddard, P. A., Singleton, J., Brambleby, J., Foronda, F., Möller, J. S., Kohama, Y., Ghannadzadeh, S., Ardavan, A., Blundell, S. J., Lancaster, T., Xiao, F., Williams, R. C., Pratt, F. L., Baker, P. J., Wierschem, K., Lapidus, S. H., Stone, K. H., Stephens, P. W., Bendix, J., Woods, T. J., Carreiro, K. E., Tran, H. E., Villa, C. J. & Manson, J. L. Antiferromagnetism in a Family of $S = 1$ Square Lattice Coordination Polymers $NiX_2(pyz)_2$ (X = Cl, Br, I, NCS; pyz = Pyrazine), *Inorg. Chem.* **55**, 3515–3529 (2016).
2. Degen, T., Sadki, M., Bron, E., König, U., Nénert, G. The HighScore suite. *Powder Diffraction*, **29(S2)**, S13-S18 (2014).
3. Bourhis, L. J., Dolomanov, O. V., Gildea, R. J., Howard, J. A. K. & Puschmann, H. The anatomy of a comprehensive constrained, restrained refinement program for the modern computing environment – Olex2 dissected. *Acta Cryst.* **A71**, 59–75 (2015).
4. Dolomanov, O. V., Bourhis, L. J., Gildea, R. J., Howard, J. A. K. & Puschmann, H. *OLEX2*: a complete structure solution, refinement and analysis program**.** *J. Appl. Cryst*. **42**, 339–341 (2009).
5. Sheldrick, G. M. Crystal structure refinement with *SHELXL Acta Cryst*. **C71**, 3–8 (2015).
6. Wilhelm, F., Jaouen, N., Rogalev, A., Stirling, W. G., Springell, R., Zochowski, S. W., Beesley, A. M., Brown, S. D., Thomas, M. F., Lander, G. H., Langridge, S., Ward, R. C. C. & Wells, M. R. X-ray magnetic circular dichroism study of uranium/iron multilayers. *Phys. Rev. B: Condens. Matter Mater. Phys*. **76**, 024425 (2007).
7. Kau, L.-S., Spira-Solomon, D. J., Penner-Hahn, J. E., Hodgson, K. O & Solomon, E. I. X-ray absorption edge determination of the oxidation state and coordination number of copper. Application to the type 3 site in Rhus vernicifera laccase and its reaction with oxygen. *J. Am. Chem. Soc.* **109**, 6433–6442 (1987).

8. Hedman, B., Hodgson, K. O. & Solomon, E. I. X-ray absorption edge spectroscopy of ligands bound to open-shell metal ions: chlorine K-edge studies of covalency in tetrachlorocuprate(2–). *J. Am. Chem. Soc.* **112**, 1643–1645 (1990).
9. Glaser, T., Hedman, B. Hodgson, K. O. & Solomon, E. I. Ligand K-Edge X-ray Absorption Spectroscopy: A Direct Probe of Ligand−Metal Covalency. *Acc. Chem. Res.* **33**, 859–868 (2000).
10. Shadle, S. E., Hedman, B., Hodgson, K. O. & Solomon, E. I. Ligand K-edge x-ray absorption spectroscopic studies: metal-ligand covalency in a series of transition metal tetrachlorides**.** *J. Am. Chem. Soc.* **117,** 2259–2272 (1995).
11. Kresse, G. & Furthmüller, J. Efficient iterative schemes for *ab initio* total-energy calculations using a plane-wave basis set. *Phys. Rev. B* **54**, 11169–11186 (1996).
12. Kresse, G. & Furthmüller, J. Efficiency of ab-initio total energy calculations for metals and semiconductors using a plane-wave basis set. *J. Comput. Mater. Sci.* **6**, 15–50 (1996).
13. Perdew, J. P., Ruzsinszky, A., Csonka, G. I., Vydrov, O. A., Scuseria, G. E., Constantin, L. A., Zhou, X. & Burke, K. Restoring the Density-Gradient Expansion for Exchange in Solids and Surfaces. *Phys. Rev. Lett.* **100**, 136406 (2009). Erratum *Phys. Rev. Lett.* **102**, 039902 (2009).
14. Dudarev, S. L., Botton, G. A., Savrasov, S. Y., Humphreys, C. J., Sutton, A. P. Electron-energy-loss spectra and the structural stability of nickel oxide: An LSDA+U study. *Phys. Rev. B* **57**, 1505 (1998).
15. Aykol, M. & Wolverton, C. Local environment dependent GGA+U method for accurate thermochemistry of transition metal compounds. *Phys. Rev. B* **90**, 115105 (2014).
16. Kresse, G. & Joubert, D. From ultrasoft pseudopotentials to the projector augmented-wave method. *Phys. Rev. B* **59**, 1758–1775 (1996).
17. Neese, F. Software update: the ORCA program system, version 4.0, *WIREs Comput. Mol. Sci.* **8**, e1327 (2018).
18. van Lenthe, E., Baerends, E. J. & Snijders, J. G. Relativistic regular two-component Hamiltonians. *J. Chem. Phys.* **99**, 4597–4610 (1993).
19. van Wüllen, C. J. Molecular density functional calculations in the regular relativistic approximation: Method, application to coinage metal diatomics, hydrides, fluorides and chlorides, and comparison with first-order relativistic calculations. *J. Chem. Phys.* **109**, 392–399 (1998).
20. Staroverov, V. N., Scuseria, G. E., Tao, J. & Perdew, J. P. Comparative assessment of a new nonempirical density functional: Molecules and hydrogen-bonded complexes. *J. Chem. Phys.* **119**, 12129–12137 (2003).
21. Weigend, F. & Ahlrichs, R. Balanced basis sets of split valence, triple zeta valence and quadruple zeta valence quality for H to Rn: Design and assessment of accuracy. *Phys. Chem. Chem. Phys.* **7**, 3297–3305 (2005).
22. Pantazis, D. A., Chen, X.-Y., Landis, C. R. & Neese, F. All-Electron Scalar Relativistic Basis Sets for Third-Row Transition Metal Atoms. *J. Chem. Theory Comput*. **4**, 908–919 (2008).

23. Yamaguchi, K., Takahara, Y. & Fueno, T. in *Applied Quantum Chemistry*; Smith, V. H., Schaefer, H. F. & Morokuma, K. Eds.; D. Reidel: Boston, 1986; p. 155.
24. Yamanaka, S., Kawakami, T., Nagao, H. & Yamaguchi, K. Effective exchange integrals for open-shell species by density functional methods. *Chem. Phys. Lett.* **231**, 25–33 (1994).
25. Humphrey, W., Dalke, A. & Schulten, K. VMD: Visual molecular dynamics. *J. Molec. Graphics* **14**, 33–38 (1996).
26. Website: http://www.ks.uiuc.eu/Researcg/vmd
27. Mott, N. F. Conduction in non-crystalline materials. *Philos. Mag.* **19**, 835–852 (1969).
28. Miller, A. & Abrahams, E. Impurity conduction at low concentrations. *Phys. Rev.* **120**, 745–755 (1960).
29. Shklovskii, B. I. & Efros , A. L. *Electronic Properties of Doped Semiconductors*, Springer-Verlag, New York, **1984**.
30. Beloborodov, I. S., Lopatin, A. V., Vinokur, V. M. & Efetov, K. B. Granular electronic systems. *Rev. Mod. Phys.* **79**, 469–518 (2007).
31. Park, J. G., Aubrey, M. L., Oktawiec, J., Chakarawet, K., Darago, L. E., Grandjean, F., Long, G. J. & Long, J. R. Charge Delocalization and Bulk Electronic Conductivity in the Mixed-Valence Metal−Organic Framework Fe(1,2,3-Triazolate)$_2$(BF$_4$)$_x$. *J. Am. Chem. Soc.* **140**, 8526−8534 (2018).
32. Xie, L. S., Sun, L., Wan, R., Park, S. S., DeGayner, J. A., Hendon, C. H. & Dincă, M. Tunable Mixed-Valence Doping toward Record Electrical Conductivity in a Three-Dimensional Metal-Organic Framework. *J. Am. Chem. Soc.* **140**, 7411−7414 (2018).
33. Aubrey, M. L., Wiers, B. M., Andrews, S. C., Sakurai, T., Reyes-Lillo, S. E., Hamed, S. M., Yu, C.-J., Darago, L. E., Mason, J. A., Baeg, J.-O., Grandjean, F., Long, G. J., Seki, S., Neaton, J. B., Yang, P. & Long, J. R. Electron Delocalization and Charge Mobility as a Function of Reduction in a Metal−Organic Framework. *Nat. Mater.* **17**, 625−632 (2018).
34. Darago, L. E., Aubrey, M. L., Yu, C. J., Gonzalez, M. I. & Long, J. R. Electronic Conductivity, Ferrimagnetic Ordering, and Reductive Insertion Mediated by Organic Mixed-Valence in a Ferric Semiquinoid Metal−Organic Framework. *J. Am. Chem. Soc.* **137**, 15703−15711 (2015).
35. DeGayner, J. A., Jeon, I.-R., Sun, L., Dincă, M. & Harris, T. D. 2D Conductive Iron-Quinoid Magnets Ordering up to T_c = 105 K via Heterogenous Redox Chemistry. *J. Am. Chem. Soc.* **139**, 4175−4184 (2017).
36. Benmansour, S., Abhervé, A., Gómez-Claramunt, P., Vallés-García, C. & Gómez-García, C. J. Nanosheets of Two-Dimensional Magnetic and Conducting Fe(II)/Fe(III) Mixed-Valence Metal−Organic Frameworks. *ACS Appl. Mater. Interfaces* **9**, 26210−26218 (2017).
37. Ziebel, M. E., Darago, L. E. & Long, J. R. Control of Electronic Structure and Conductivity in Two-Dimensional Metal−Semiquinoid Frameworks of Titanium, Vanadium, and Chromium. *J. Am. Chem. Soc.* **140**, 3040−3051 (2018).

38. Pedersen, K. S., Perlepe, P., Aubrey, M. L., Woodruff, D. N., Reyes-Lillo, S. E., Reinholdt, A., Voigt, L., Li, Z., Borup, K., Rouzières, M., Samohvalov, D., Wilhelm, F., Rogalev, A., Neaton, J. B., Long, J. R. & Clérac, R. Formation of the layered conductive magnet $CrCl_2(pyrazine)_2$ through redox-active coordination Chemistry. *Nat. Chem.* **10**, 1056−1061 (2018).
39. Bhattacharya, B., Layek, A., Mehboob Alam, M., Maity, D. K., Chakrabarti, S., Ray, P. P. & Ghoshal, D. Cd(II) Based Metal−Organic Framework Behaving as a Schottky Barrier Diode. *Chem. Commun.* **50**, 7858−7861 (2014).
40. Qu, L., Iguchi, H., Takaishi, S., Habib, F., Leong, C. F., D'Alessandro, D. M., Yoshida, T., Abe, H., Nishibori, E. & Yamashita, M. Porous Molecular Conductor: Electrochemical Fabrication of Through-Space Conduction Pathways among Linear Coordination Polymers. *J. Am. Chem. Soc.* **141**, 6802−6806 (2019).
41. Mähringer, A., Döblinger, M., Hennemann, M., Gruber, C., Fehn, D., Scheurle, P. I., Hosseini, P., Santourian, I., Schirmacher, A., Rotter, J. M., Wittstock, G., Meyer, K., Clark, T., Bein, T. & Medina, D. D. An Electrically Conducting Three-Dimensional Iron–Catecholate Porous Framework. *Angew. Chem. Int. Ed*. **60**, 18065–18072 (2021).
42. Dixon, M., Hoare, F. E., Holden, T. M. & Moody, D. E. The low temperature specific heats of some pure metals (Cu, Ag, Pt, Al, Ni, Fe, Co). *Proc. R. Soc. Lond.* **A285**, 561–580 (1965).
43. Loram, J. W., Mirza, K. A., Wade, J. M., Cooper, J. R. & Liang, W. Y. The electronic specific heat of cuprate superconductors. *Physica C: Superconductivity* **235-240**, 134–137 (1994).
44. Mackenzie, A. P., Ikeda, S., Maeno, Y., Fujita, T., Julian, S. R. & Lonzarich. G. G. The Fermi Surface Topography of Sr_2RuO_4. *J. Phys. Soc. Jpn.* **67**, 385–388 (1998).
45. Vignolle, B., Carrington, A., Cooper, R. A., French, M. M. J., Mackenzie, A. P., Jaudet, C., Vignolles, D., Proust, C. & Hussey, N. E. Quantum oscillations in an overdoped high-T_C superconductor. *Nature* **455,** 952–955 (2008).
46. Mackenzie, A. P., Julian, S. R., Diver, A. J., McMullan, G. J., Ray, M. P., Lonzarich, G. G., Maeno, Y., Nishizaki, S. & Fujita, T. Quantum Oscillations in the Layered Perovskite Superconductor Sr_2RuO_4. *Phys. Rev. Lett.* **76**, 3786–3789 (1996).
47. Andraka, B., Stewart, G. R., Carlson, K. D., Wang, H. H., Vashon, M. D. & William, J. M. Specific heat in zero and applied magnetic fields of the organic superconductor α-di[bis(ethylenedithio) tetrathiafulvalene]-ammonium-tetra(thiocyanato)mercurate [α-$(ET)_2(NH_4)Hg(SCN)_4$]. *Phys. Rev. B* **42**, 9963–9966 (1990).
48. Andraka B., Kim, J. S., Stewart, G. R., Carlson, K. D., Wang, H. H., Williams, J. M. Specific heat in high magnetic field of k-di[bis(ethylenedithio) tetrathiafulvalene)-di(thiocyano) cuprate [k-$(ET)_2Cu(NCS)_2$): Evidence for strong-coupling superconductivity. *Phys. Rev. B* **40**, R11345–11347 (1989).

49. Andraka, B., Jee, C. S., Kim, J. S., Stewart, G. R., Carlson, K. D., Wang, H. H., Crouch, A. V. S., Kini, A. M. & Williams, J. M. Specific heat of the high T_C organic superconductor κ-$(ET)_2Cu[N(CN)_2]Br$. *Solid State Commun.* **79**, 57–59 (1991).

50. Wosnitza, J., Liu, X., Schweitzer, D. & Keller, H. J. Specific heat of the organic superconductor κ-$(BEDT\text{-}TTF)_2I_3$. *Phys. Rev. B* **50**, 12747–12751 (1994).

51. Stewart, G. R., O'Rourke, J., Crabtree, G. W., Carlson, K. D., Wang, H. H., Williams, J. M., Gross, F. & Andres, K. Specific heat of the ambient-pressure organic superconductor β-di[bis(ethylenedithio)tetrathiafulvalene] triiodide [β-$(BEDT\text{-}TTF)_2I_3$]. *Phys. Rev. B* **33**, R2046–2048 (1986).

52. Wilson, K. G. The renormalization group: Critical phenomena and the Kondo problem. *Rev. Mod. Phys*. **47**, 773–840 (1975).

53. Ikeda, S. I., Maeno, Y., Nakatsuji, S., Kosaka, M. & Uwatoko, Y. Ground state in $Sr_3Ru_2O_7$: Fermi liquid close to a ferromagnetic instability. *Phys. Rev. B* **62**, R6089–6092 (2000).

54. McKenzie, R. H. Wilson's ratio and the spin splitting of magnetic oscillations in quasi-two-dimensional metals. *arXiv:cond-mat/9905044v2* (1999).
