# Supplementary material for: From an antiferromagnetic insulator to a strongly correlated metal in square-lattice MCl2(pyrazine)2 coordination solids
Source: Nat Commun. 2022 Sep 30;13:5766. doi: 10.1038/s41467-022-33342-5 (PMC9525593; doi:10.1038/s41467-022-33342-5)
Supplement: Supplementary file 6 — Description of Additional Supplementary Files [file 41467_2022_33342_MOESM6_ESM.pdf]

Title: Supplementary Dataset 1:
Description: CIF file for $VCl_2(pyz)_2$ from powder diffraction data

Title: Supplementary Dataset 2:
Description: CIF file for $TiCl_2(pyz)_2$ from powder diffraction data

Title: Supplementary Dataset 3:
Description: CIF file for $VCl_2(py)_4$ from single-crystal diffraction data

Title: Supplementary Dataset 4:
Description: CIF file for $VCl_2(pyz)_2$ from single-crystal diffraction data
